# Characteristics of male perpetrators of intimate partner violence and implications for women’s HIV status: A pooled analysis of cohabiting couples from 27 countries in Africa (2000–2020)

**DOI:** 10.1371/journal.pgph.0002146

**Published:** 2023-09-06

**Authors:** Salome Kuchukhidze, Dimitra Panagiotoglou, Marie-Claude Boily, Souleymane Diabaté, Jeffrey W. Imai-Eaton, Heidi Stöckl, Francisco Mbofana, Rhoda K. Wanyenze, Mathieu Maheu-Giroux

**Affiliations:** 1 Department of Epidemiology and Biostatistics, School of Population and Global Health, McGill University, Montréal, QC, Canada; 2 MRC Centre for Global Infectious Disease Analysis, School of Public Health, Imperial College London, London, United Kingdom; 3 Département de Médecine Sociale et Préventive, Université Laval, Québec, QC, Canada; 4 Centre de recherche du CHU de Québec-Université Laval, Québec, QC, Canada; 5 Département de Médecine et Spécialités Médicales, Université Alassane Ouattara, Bouaké, Côte d’Ivoire; 6 Center for Infectious Disease Dynamics, Department of Epidemiology, Harvard T.H. Chan School of Public Health, Boston, MA, United States of America; 7 Institute for Medical Information Processing, Biometry, and Epidemiology, Medical Faculty, LMU Munich, Munich, Germany; 8 Conselho Nacional de Combate ao HIV/Sida, Maputo, Mozambique; 9 Department of Disease Control and Environmental Health, School of Public Health, College of Health Sciences, Makerere University, Kampala, Uganda; University of Manitoba College of Medicine: University of Manitoba Max Rady College of Medicine, CANADA

## Abstract

Intimate partner violence (IPV) may increase women’s HIV acquisition risk. Still, knowledge on pathways through which IPV exacerbates HIV burden is emerging. We examined the individual and partnership-level characteristics of male perpetrators of physical and/or sexual IPV and considered their implications for women’s HIV status. We pooled individual-level data from nationally representative, cross-sectional surveys in 27 countries in Africa (2000–2020) with information on past-year physical and/or sexual IPV and HIV serology among cohabiting couples (≥15 years). Current partners of women experiencing past-year IPV were assumed to be IPV perpetrators. We used Poisson regression, based on Generalized Estimating Equations, to estimate prevalence ratios (PR) for male partner and partnership-level factors associated with perpetration of IPV, and men’s HIV status. We used marginal standardization to estimate the adjusted risk differences (aRD) quantifying the incremental effect of IPV on women’s risk of living with HIV, beyond the risk from their partners’ HIV status. Models were adjusted for survey fixed effects and potential confounders. In the 48 surveys available from 27 countries (N **=** 111,659 couples), one-fifth of women reported that their partner had perpetrated IPV in the past year. Men who perpetrated IPV were more likely to be living with HIV (aPR = 1.09; 95%CI: 1.01–1.16). The aRD for living with HIV among women aged 15–24 whose partners were HIV seropositive and perpetrated past-year IPV was 30% (95%CI: 26%-35%), compared to women whose partners were HIV seronegative and did not perpetrate IPV. Compared to the same group, aRD among women whose partner was HIV seropositive without perpetrating IPV was 27% (95%CI: 23%-30%). Men who perpetrated IPV are more likely to be living with HIV. IPV is associated with a slight increase in young women’s risk of living with HIV beyond the risk of having an HIV seropositive partner, which suggests the mutually reinforcing effects of HIV/IPV.

## Introduction

Ending violence against women is a public health priority globally. IPV has serious short and long-term physical and mental health consequences, including injury, depression, anxiety, unwanted pregnancies, and sexually transmitted infections [1]. Several prospective [2, 3] and population-based studies [4, 5] suggested that intimate partner violence (IPV) also contributes to HIV risk. One in three women between the ages of 15 and 49 in sub-Saharan Africa–the region with the highest HIV burden–have experienced physical and/or sexual IPV in their lifetime [1]. In the same group, 20% of total years of life lost as a result of disability is attributable to HIV [6]. Adolescent girls and young women (AGYW), who are the most vulnerable to IPV, are three times as likely to acquire HIV and twice as likely to be living with HIV than men of the same age [7, 8]. As part of the new strategy to end AIDS, the *2021 United Nations General Assembly (UNGA)* adopted a *Political Declaration on HIV and AIDS* which committed to reducing the proportion of women and girls who experience gender-based violence to less than 10% by 2025 [9]. Improving understanding of the factors and pathways associated with male-perpetrated IPV and their implications for women’s HIV acquisition risk is important to meet this commitment.

Studies that examined links between IPV and HIV have focused on the characteristics of women experiencing IPV [2–5]. However, there is increased evidence that pathways between IPV and HIV are also influenced by male partner characteristics. Distal determinants of HIV acquisition, such as male’s concurrent sexual partnerships [10, 11], participation in transactional sex [11], and inconsistent condom use [12] could increase men’s risk of HIV acquisition and, ultimately, women’s risk of acquisition [13]. These sexual behaviors and subsequent HIV risks are shown to be more common among men who perpetrate IPV, which may arise from the underlying, dominant ideals of masculinity [12, 14, 15]. Proximal determinants such as men’s HIV seropositivity, and partnership characteristics could further compound women’s risk. For instance, disparities in couples’ age, education and earnings, which often disproportionately affect AGYW [16], may inhibit women’s decision-making power on circumstances around sex and elevate the risk of HIV acquisition if their male partner has an unsuppressed viral load.

The links between IPV, sexual behaviors, and HIV are rooted in the social norms that perpetuate gender inequality. Studies have shown that masculine norms around virility and resilience may shape sexual behaviors and subsequent risk of HIV acquisition in men [17, 18]. Attitudes on relationship power dynamics, which underly IPV perpetration, limit women’s sexual agency, thus contributing to their HIV risk.

Several population-based studies linked women’s experience of IPV with HIV. However, few considered their male partners’ HIV status, men’s engagement in the HIV care cascade, or sexual behaviors [2, 4]. Those that do, are cross-sectional and often limited by small sample sizes of cohabiting couples [5]. Conversely, multi-country studies describing characteristics of male perpetrators of IPV have not used an HIV lens and did not seek to understand the transmission risk to their female partner [19–21]. Using information on cohabiting male-female dyads from population-based surveys could help fill these knowledge gaps.

The aim of this study is to describe the characteristics of men perpetrating physical and/or sexual IPV and investigate how these characteristics impact women’s HIV status among cohabiting couples in select African countries. We achieve this by leveraging available nationally representative, cross-sectional population-based surveys with information on both IPV and HIV. Specifically, we address three research questions. First, what male partner and partnership-level characteristics are associated with IPV? Second, are men who are reported to perpetrate IPV more likely to report behaviors that increase their risk of HIV acquisition and to be living with HIV? Third, does experiencing IPV increase young women’s risk of living with HIV, beyond the risk associated with their male partner’s HIV status?

## Methods

### Ethics statement

Deidentified participant data were used in the study. Ethics approval was obtained from the institutional review board of the Faculty of Medicine and Health Sciences at McGill University (Montréal, QC, Canada; approval number A12-B95–21B).

### Data sources and study population

We reviewed available nationally representative, cross-sectional surveys conducted in 27 countries in Africa between 2000 and 2020 with available respondent-level data on IPV and HIV testing, as described by Kuchukhidze and colleagues [5]. The included countries were in the geographic region of sub-Saharan Africa; the classification of this region and sub-regions was aligned with that of the United Nations Statistics Division [22]. The study population comprised currently cohabiting, married or partnered women and men (≥15 years) that participated in the *Demographic and Health Surveys* (DHS), *AIDS Indicator Survey* (AIS), and *Population-based HIV Impact Assessment* (PHIA) surveys. PHIA, DHS and AIS used a stratified, two-stage, household-based cluster sampling design. Survey instruments included household questionnaires, individual questionnaires, and collection of biomarkers. Individual interviews included adult women and men, aged ≥15 years with slight variations in the upper age limit for eligibility across the surveys [23, 24]. To create the analytical sample, we used data from survey participants who mutually declared to be married or co-habiting at the time of the survey and in which the female partner completed the IPV survey questionnaire.

In PHIA, data on past-year IPV were collected from one randomly selected woman in each household and, in DHS, from all women in a fraction of households (usually one third). For DHS, we used the couple’s dataset; for PHIA, a dataset of cohabiting men and women was linked based on an identifier corresponding to the household member confirmed as the person’s partner. All included surveys allowed for this linkage to identify unique, partnered couples.

### Definitions and measurement

Perpetration of physical and/or sexual IPV over the past year among cohabitating couples was defined based on the women’s self-reported experience of IPV, which was defined as the experience of physical and/or sexual violence in the past year by a current or former male intimate partner in the context of marriage or cohabitation. Current partners of women experiencing IPV in the past year were assumed to be perpetrators of IPV [13, 25]. From here onwards, when referring to “perpetrators” of IPV, we refer to men whose female partner reported experiencing IPV in the past year.

Perpetration of physical and/or sexual IPV over the past year, as opposed to lifetime, was used for two main reasons. First, it would not be possible to link lifetime reports of IPV to a specific partner since the couple may have ceased cohabiting. Second, past-year reports match the timeframe for sexual behaviors reported in the surveys (e.g., condom use, payment for sex, number of sex partners in the past year). We combined physical and sexual IPV in a single measure given the considerable overlap between the two [5]. All surveys used an acts-specific instrument, based on the modified Conflict Tactics Scale [26], to collect information on IPV (Table A in S1 Text).

Potential factors correlated with IPV pertained to male individual factors and partnership-level factors. Individual factors included: accepting attitudes on IPV, alcohol use frequency, and polygyny defined as having more than one wife/cohabiting partner. Partnership factors included: couple age disparity, earning disparity, women’s say in household decision-making, and household headship (male/female). Couple age disparity was defined as the age difference between the man and the woman in the partnership. Earning disparity measured whether a woman earned more, less than, or the same as her partner, per survey questionnaire. DHS defined household headship as the person considered responsible for the household [27].

Self-reported factors for men’s risk of living with HIV included: payment for sex in the past year, condom use at last sex with the most recent partner in the past year, number of sex partners in the past year, and point-prevalence of concurrency defined as having more than one sexual partnership at a single point in time six months before the interview. Definition of concurrency was aligned with the primary indicator recommended by the UNAIDS Reference Group on Estimates, Modelling and Projections Working Group on Measuring Concurrent Sexual Partnerships [28]. All variables were extracted from individual participant surveys, and survey questions and measurements were generally consistent across the surveys.

HIV seropositivity was measured among consenting male and female participants at the time of survey administration via enzyme-linked immunosorbent assay (ELISA). The Zambia 2013–14 DHS was excluded from all analyses using HIV seropositivity due to concerns about its reliability [29].

### Data analysis

To describe characteristics of male IPV perpetrators, individual-level data from each survey were pooled to calculate crude and adjusted prevalence ratios (PR) of the association between the male and partnership-level variables and the IPV outcome. First, univariable Poisson regression models based on Generalized Estimating Equations (GEE) with robust standard errors and clustering by primary sampling unit (PSU) without survey weights [30], were used to calculate crude estimates. Multivariable models were adjusted for basic socio-demographic variables: male age (five-year age groups to account for the non-linear age effect), household wealth quintile, residence type (rural, urban), and male education (none, primary, secondary, higher). Survey-level fixed effects were included in the adjusted models to account for unmeasured survey-level confounders.

For the second objective, IPV was the primary independent variable since we sought to identify if male perpetrators of IPV were more likely to report selected sexual behaviors (condom use in the past year, payment for sex in the past year, number of sex partners in the past year, concurrency of multiple sexual partners) and to be living with HIV. Here, we treated IPV as an independent variable since male sexual behaviors might be confounded by the underlying, unmeasured patriarchal attitudes that grant men a sexual prerogative [31]. As in the analyses above, multivariable models were adjusted for male demographic characteristics and survey identifier. For the male seropositivity outcome, we additionally adjusted for men’s lifetime number of sexual partners (Table B in S1 Text).

For the final objective, we used effect measure modification analysis to assess if IPV modified women’s absolute risk of living with HIV. We restricted this analysis to adolescent girls and young women aged 15–24 years for two reasons. First, we aimed to estimate the additional HIV risk due to IPV in the subgroup of women with the highest IPV prevalence and HIV incidence. Second, older women are more likely to have lived with HIV for longer due to higher HIV incidence in younger age groups. Therefore, past-year IPV is more likely to have preceded HIV acquisition among women aged 15–24 years. The analysis on all women is reported in the Text A in S1 Text.

We used marginal standardization based on GEE with robust standard errors. Crude and adjusted risk differences between ‘doubly exposed’ (women whose male partner lived with HIV and perpetrated IPV in the past year) and ‘doubly unexposed’ (the reference category–women whose male partner did not live with HIV and did not perpetrate IPV) were calculated. Risk differences between ‘singly exposed’ (women whose partner perpetrated IPV only, or whose partner lived with HIV only) and ‘doubly unexposed’ were also obtained. We estimated 95% confidence intervals (Cis) calculated via bootstrapping, where the resampling unit was the primary sampling unit. The model was adjusted for female demographic characteristics (linear age effect for analysis specific to 15–24 year-old women, and categorical five-year age groups in all women), household wealth, residence, type of education, women’s lifetime number of sex partners (1, 2, ≥3) and survey-level fixed effects (Table B in S1 Text). Men’s sexual behaviors were not included in the adjustment since they would affect women’s HIV risk primarily through male HIV status, which was adjusted for in our analyses.

We calculated the *Relative Excess Risk due to Interaction* (RERI) to understand the presence of additive effect measure modification between male HIV seropositivity and male perpetrated IPV (Table B in S1 Text) [32]. To quantify the magnitude of EMM under an additive model, we calculated the difference between the expected joint effect of male HIV status and IPV perpetration (the sum of their unique effects) and their observed joint effects. Further methodological detail and equations are in Text A in S1 Text.

Additionally, we estimated the association between past-year IPV perpetration and ART uptake and viral load suppression among HIV seropositive male partners in the small subset of surveys with this information. These analyses were adjusted for male demographic characteristics (five-year age group, wealth quintile, education, residence), male frequency of alcohol consumption (never, sometimes, often) due to its links with both IPV and male engagement in HIV care cascade [33], and survey identifier.

### Sensitivity analyses

First, we explored the heterogeneity of effect size estimates across survey for each model by calculating survey-specific crude prevalence ratios and pooling them using both fixed and random-effect meta-analyses. We conducted subgroup (moderator) analyses by survey region and/or year when heterogeneity was moderate (25%-50%) to high (>50%) [34]. Second, we also calculated crude and adjusted prevalence ratios stratified by region. Third, we excluded women who had two or more sexual partners in the past year to reduce the likelihood that women’s reports of experiencing IPV in the past year referred to someone other than their current, cohabiting partner.

If either men or women respondents declined survey participation, were away from the household at the time of the interview, or if their identification of a cohabiting partner did not match their partner’s report, the couple was excluded from the study sample. Survey participation could be associated with both IPV and HIV status in men, leading to selection bias. In a probabilistic sensitivity analysis, we assumed selection probabilities which were assigned to perpetrators and non-perpetrators with and without the outcome of interest (HIV seropositivity) based on the existing literature (Text B in S1 Text). We repeatedly resampled these selection probabilities from a uniform distribution to adjust the observed crude prevalence ratio for potential differential participation [35]. We estimated the median value of the simulations as a bias-corrected crude prevalence ratio and calculated the 95% uncertainty intervals.

## Results

### Description of included surveys and the study population

**Fig 1:**
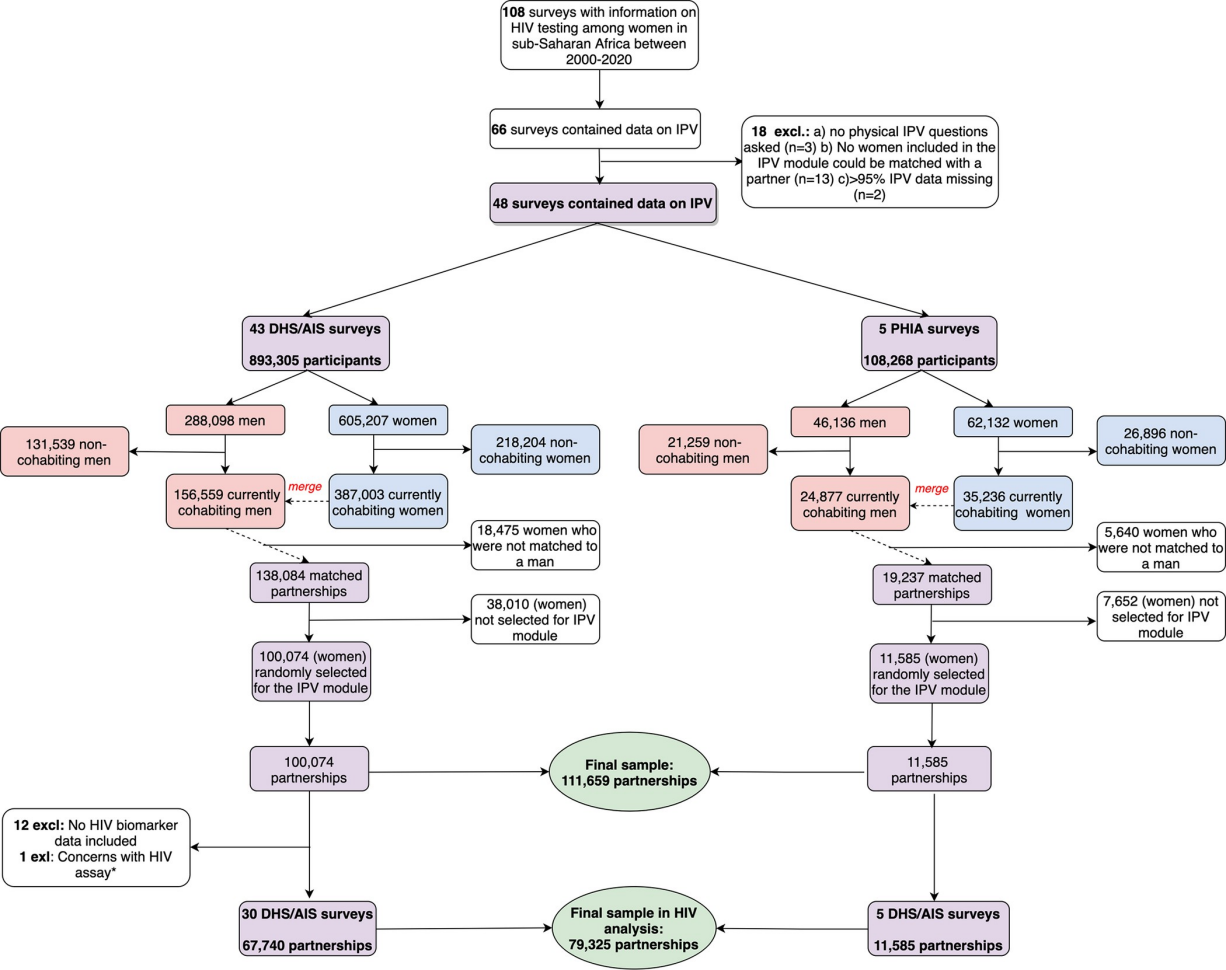
A flowchart describing the steps taken to create the final, analytical sample of cohabiting couples and the number of individuals included in the HIV analyses from each survey type. *The Zambia 2013–14 DHS was excluded from the analyses of HIV seropositivity due to concern about the reliability of the HIV testing algorithm assay. (AIS = AIDS Indicator Survey; DHS = Demographic and Health Survey; Excl. = excluded; IPV = intimate partner violence; PHIA = Population-based HIV Impact Assessment Survey).

We identified 108 nationally representative surveys with data on HIV, of which 66 also had data on IPV. Eighteen surveys were excluded due to physical IPV questions not being asked, IPV data missingness, or women in couples’ dataset not selected for the IPV module, resulting in 48 surveys from 27 countries. These surveys included 1,001,573 female and male participants, 89% from DHS/AIS. Among these, 181,436 men and 422,239 women were cohabiting at the time of the survey. Linking female with male datasets resulted in 157,321 partnerships. Thus, 87% (157,321/181,436) of men were successfully matched to their female partner. Among these couples, 111,659 had been randomly selected for the IPV module and included as the final dataset (Fig 1). Since the IPV module was administered to only one randomly selected woman in the household, men in polygynous partnerships were linked to the partner who participated in the IPV module. HIV biomarkers were collected in all five PHIA and 70% (30/43) of DHS surveys; hence 79,325 partnerships were included in HIV analyses. Ten countries had more than one survey. The median year of data collection was 2013. Most surveys were from Eastern Africa (54%) and the least from Southern Africa (6%).

### Characteristics of the study population

Overall, 21% of women reported that their male partners had perpetrated IPV in the past year, ranging from 25% in Central Africa to 6% in Southern Africa (Table C in S1 Text). Men who perpetrated past-year IPV, and their female partners who experienced it, were younger, more likely to have only primary education, and were less wealthy compared to those who did not (Table 1). Among couples where men were reported to perpetrate IPV, women were less likely to have a say in household decision-making (41%) compared to where it was not (47%). Among men who perpetrated IPV, more had accepting attitudes on IPV (34% versus 25%). Men who perpetrated IPV were more likely to consume alcohol “*Often*” (18%) and “*Sometimes*” (29%) compared to those did not (6% and 18%, respectively). Age difference between partners was comparable between couples where men perpetrated IPV (6.2 years) versus where they did not (6.9 years) (Table 1).

**Table 1:** Summary of individual and partnership-level characteristics among men and women who are currently cohabiting, stratified by perpetration or experience of past-year physical and/or sexual intimate partner violence (IPV).

		Past year physical and/or sexual IPV, n(%)			
		Male		Female	
	**N**_**survey**_	**Yes (N**_**ind**_ **= 23,777)**	**No (N**_**ind**_ **= 86,596)**	**Yes (N**_**ind**_ **= 23,777)**	**No (N**_**ind**_ **= 86,596)**
**Demographic characteristics, n (%)**					
**Age, n (%)**	**48**				
15–24 years		1,745 (7.3%)	5,490 (6.3%)	6,638 (27.9%)	23,288 (26.9%)
25–34 years		9,719 (40.9%)	30,527 (35.3%)	10,943 (46.0%)	37,059 (42.8%)
35–44 years		7,976 (33.5%)	29,942 (34.6%)	5,105 (21.5%)	20,347 (23.5%)
45–64 years		4,329 (18.2%)	20,106 (23.2%)	1,089 (4.6%)	5,674 (6.6%)
65+ years		8 (0.0%)	531 (0.6%)	2 (0.0%)	228 (0.3%)
*(Missing)*		0 (0%)	0 (0%)	0 (0%)	0 (0%)
**Education, n (%)**	**48**				
None		4,788 (20.1%)	21,158 (24.4%)	6,705 (28.2%)	28,813 (33.3%)
Primary		10,191 (42.9%)	31,181 (36.0%)	11,062 (46.5%)	32,516 (37.5%)
Secondary		7,697 (32.4%)	26,995 (31.2%)	5,594 (23.5%)	21,631 (25.0%)
Higher		1,100 (4.6%)	7,245 (8.4%)	412 (1.7%)	3,626 (4.2%)
*(Missing)*		1 (0.0%)	17 (0.0%)	4 (0.0%)	10 (0.0%)
**Recent employment, n (%)**	**48**				
Employed		22,665 (95.3%)	78,603 (90.8%)	16,751 (70.5%)	52,736 (60.9%)
Unemployed		1,108 (4.7%)	7,972 (9.2%)	7,021 (29.5%)	33,836 (39.1%)
*(Missing)*		4 (0.0%)	21 (0.0%)	5 (0.0%)	24 (0.0%)
**Household characteristics, n (%)**		**Yes (N**_**ind**_ **= 23,777)**		**No (N**_**ind**_ **= 86,596)**	
**Wealth quintile, n (%)** ^**^	**48**				
Lowest		5,531 (23.3%)		18,970 (21.9%)	
Second lowest		5,380 (22.6%)		18,161 (21.0%)	
Middle		5,064 (21.3%)		17,213 (19.9%)	
Second highest		4,626 (19.5%)		16,417 (19.0%)	
Highest		3,176 (13.4%)		15,815 (18.3%)	
*(Missing)*		0 (0.0%)		20 (0.0%)	
**Residence type, n (%)** ^**^	**48**				
Rural		17,049 (71.7%)		60,504 (69.9%)	
Urban		6,728 (28.3%)		26,092 (30.1%)	
*(Missing)*		0 (0%)		0 (0%)	
**HIV and behavioral risk factors, n (%)**					
**HIV prevalence, n (%)**	**35**	**Yes (N**_**ind**_ **= 16,364)**	**No (N**_**ind**_ **= 55,745)**	**Yes (N**_**ind**_ **= 16,364)**	**No (N**_**ind**_ **= 55,745)**
Yes		1,137 (6.9%)	4,082 (7.3%)	1,221 (7.5%)	4,115 (7.4%)
No		13,666 (83.5%)	46,267 (83.0%)	14,146 (86.4%)	47,749 (85.7%)
*(Missing)*		1,561 (9.5%)	5,396 (9.7%)	997 (6.1%)	3,881 (7.0%)0
**Condom use at last sex with most recent partner in the past 12 months, n (%)**	**48**	**Yes (N**_**ind**_ **= 23,777)**	**No (N**_**ind**_ **= 86,596)**	**Yes (N**_**ind**_ **= 23,777)**	**No (N**_**ind**_ **= 86,596)**
Yes		1,977 (8.3%)	6,856 (7.9%)	1,102 (4.6%)	4,238 (4.9%)
No		21,241 (89.3%)	76,409 (88.2%)	21,848 (91.9%)	78,473 (90.6%)
*(Missing)*		559 (2.4%)	3,331 (3.8%)	827 (3.5%)	3,885 (4.5%)
**Number of sex partners in the past 12 months, n (%)**	**47**	**Yes (N**_**ind**_ **= 23,391)**	**No (N**_**ind**_ **= 85,704)**	**Yes (N**_**ind**_ **= 23,391)**	**No (N**_**ind**_ **= 85,704)**
1		18,411 (78.7%)	70,465 (82.2%)	22,920 (98.0%)	84,494 (98.6%)
2+		4,809 (20.6%)	14,260 (16.6%)	357 (1.5%)	582 (0.7%)
*(Missing)*		171 (0.7%)	979 (1.1%)	114 (0.5%)	628 (0.7%)
**Point prevalence of concurrency,**^¤^ **n (%)**	**47**	**Yes (N**_**ind**_ **= 4,809)**	**Yes (N**_**ind**_ **= 14,260)**	**Yes (N**_**ind**_ **= 357)**	**Yes (N**_**ind**_ **= 582)**
Yes		1,878 (39.1%)	6,771 (47.5%)	89 (24.9%)	113 (19.4%)
No		2,931 (60.9%)	7,489 (52.5%)	268 (75.1%)	469 (80.6%)
*(Missing)*		0 (0.0%)	(0.0%)	(0.0%)	(0.0%)
**Male payment for sex in past 12 months, n (%)** ^*^	**43**	**Yes (N**_**ind**_ **= 22,216)**	**No (N**_**ind**_ **= 81,929)**		
Yes		621 (2.8%)	1,592 (1.9%)	**-**	**-**
No		21,276 (95.8%)	78,001 (95.2%)	**-**	**-**
*(Missing)*		319 (1.4%)	2,336 (2.9%)	**-**	**-**
**Male individual predictors of IPV, n (%)**					
**Man has more than one cohabiting partner, n (%)**	**48**	**Yes (N**_**ind**_ **= 23,777)**	**No (N**_**ind**_ **= 86,596)**		
Yes		2,974 (12.5%)	10,580 (12.2%)	**-**	**-**
No		20,791 (87.4%)	75,986 (87.7%)	**-**	**-**
*(Missing)*		12 (0.1%)	30 (0.0%)	**-**	**-**
**Male accepting attitudes on IPV, n (%)** ^**‡**^	**43**	**Yes (N**_**ind**_ **= 23,093)**	**No (N**_**ind**_ **= 77,549)**	**-**	**-**
Yes		7,758 (33.6%)	18,982 (24.5%)	**-**	**-**
No		15,066 (65.2%)	57,646 (74.3%)	**-**	**-**
*(Missing)*		269 (1.2%)	921 (1.2%)	**-**	**-**
**Male alcohol use frequency, n (%)** ^**§**^	**46**	**Yes (N**_**ind**_ **= 22,480)**	**No (N**_**ind**_ **= 84,834)**	**-**	**-**
Never		11,744 (52.2%)	62,948 (74.2%)	**-**	**-**
Sometimes		6,619 (29.4%)	15,574 (18.4%)	**-**	**-**
Often		4,063 (18.1%)	4,814 (5.7%)	**-**	**-**
*(Missing)*		54 (0.2%)	1,498 (1.8%)	**-**	**-**
**Partnership predictors of IPV**					
**Couple earning disparity, n (%)** ^**^	**43**	**Yes (N**_**ind**_ **= 23,229)**		**No (N**_**ind**_ **= 76,026)**	
Woman less than man		7,303 (31.4%)		24,620 (32.4%)	
About the same		1,362 (5.9%)		4,097 (5.4%)	
Woman more than man		966 (4.2%)		2,471 (3.3%)	
Woman not received cash earnings in the past year		12,630 (54.4%)		41,602 (54.7%)	
*(Missing)*		968 (4.2%)		3,236 (4.3%)	
**Couple age disparity, mean(sd)** ^**^	**48**	6.19 (5.48)		6.94 (5.66)	
**Woman has a say in household decision-making, n (%)**^**†**^ ^**^	**48**	**Yes (N**_**ind**_ **= 23,777)**		**No (N**_**ind**_ **= 86,596)**	
Yes		9,779 (41.1%)		40,244 (46.5%)	
No		13,977 (58.8%)		46,264 (53.4%)	
*(Missing)*		21 (0.1%)		88 (0.1%)	
**Household head, n (%)** ^**^	**48**	**Yes (N**_**ind**_ **= 23,777)**		**No (N**_**ind**_ **= 86,596)**	
Female		1,114 (4.7%)		4,480 (5.2%)	
Male		22,663 (95.3%)		82,116 (94.8%)	

IPV = Intimate Partner Violence; N_survey_ = Number of surveys; N_ind_ = Number of individuals; sd = standard deviation.

**†** In PHIA surveys the indicator on household decision making is comprised of two variables: *healthcare decision making* and *decision making on household spending*. To harmonize the definitions between PHIA and DHS (comprised of *healthcare decision making*, *decision making on visits to family/relatives* and *decision making on large household purchases*), we removed *household spending* from the definition of the composite covariate in PHIA surveys. Thus, in PHIA the indicator on household decision making is only reflective of women’s *healthcare decision-making*.

**‡** In PHIA surveys included in this analysis, the definition of “accepting attitudes on IPV” indicator does not include a question on whether they agree or disagree that *wife-beating is justified if wife burns food*.

§ The denominator for Zimbabwe DHS 2005 survey includes only women who had ever experienced IPV, hence those who had not, would be coded as missing. Furthermore, in PHIA surveys this question is asked to the individual respondents (both men and women) while it is asked to women in reference to their male partner in DHS surveys.

¤ Denominator includes women and men who had two or more sexual partners in the past year. Study sample in Gambia 2013 DHS survey included women who only had one or no sex partners in the past year which is why this survey was not included for concurrency summary estimate among women. This explains different denominators among men and women.

* Not collected in the women’s survey

** Partnership/household level characteristics, thus the same for both men and women

Perpetrators of IPV were only slightly more likely to report paying for sex in the past year (3%) compared to non-perpetrators (2%). A higher proportion of perpetrators (21%) had two or more sex partners in the past year compared to non-perpetrators (17%). Women who reported that their partner perpetrated IPV were more likely to have concurrent sex partners (25%) compared to those whose did not (19%). Crude HIV prevalence was comparable between perpetrators (7%) and non-perpetrators of IPV (7%) (Table 1).

### Variables correlated with past-year physical and/or sexual intimate partner violence perpetration

In adjusted analyses, male accepting attitudes on IPV (aPR = 1.24; 95%CI: 1.21–1.27), frequent alcohol use (aPR = 2.90; 95%CI: 2.81–2.99), and being in a polygynous partnership (aPR = 1.17; 95%CI: 1.13–1.21) were associated with IPV perpetration in the past year (Table 2).

**Table 2:** Crude and adjusted prevalence ratios of the association between partnership and male individual characteristics and perpetration of past year physical and/or sexual intimate partner violence.

Partnership characteristics	N_survey_	N_ind_	Crude prevalence ratio (95%CI)	Adjusted prevalence ratio (95%CI)†
**Couple earning disparity**	43	95,051		
Less than him			Referent	Referent
Same			0.98 (0.93, 1.04)	0.89 (0.84, 0.93)
More than him			1.14 (1.07, 1.21)	1.12 (1.06, 1.18)
Woman not paid in cash/kind			0.98 (0.96, 1.01)	0.91 (0.89, 0.94)
**Couple age disparity**	48	110,373	0.99 (0.99, 0.99)	1.00 (1.00, 1.00)
**Woman has a say in household decision-making**	48	110,264		
Yes			0.78 (0.76, 0.80)	0.82 (0.80, 0.84)
No			Referent	Referent
**Household head**	48	110,373		
Female			0.87 (0.82, 0.93)	0.96 (0.91, 1.01)
Male			Referent	Referent
**Partnership predictors of IPV**				
**Male accepting attitudes on IPV**	43	99,452		
Yes			1.33 (1.3, 1.37)	1.24 (1.21, 1.27)
No			Referent	Referent
**Man has more than one wife/cohabiting partner**	48	110,331		
Yes			1.11 (1.07, 1.15)	1.17 (1.13, 1.21)
No			Referent	Referent
**Male alcohol use frequency**				
Never	46	105,762	Referent	Referent
Sometimes			1.80 (1.74, 1.85)	1.77 (1.72, 1.82)
Often			2.81 (2.72, 2.90)	2.90 (2.81, 2.99)

IPV = intimate partner violence; N_ind_ = Number of individuals in the adjusted analyses; N_survey_ = Number of surveys in the adjusted analyses; PR = Prevalence Ratio.

† All models are adjusted for male age (five-year age groups), male education (none, primary, secondary, higher), wealth quantile, residence type (rural, urban), survey identifier.

In partnerships where women earned more than men, women were 12% more likely to report experiencing IPV (aPR = 1.12; 95%CI: 1.06–1.18), though when women had decision-making power in the household, men were less likely to perpetrate IPV (aPR = 0.82; 95%CI: 0.80–0.84). In households headed by women, IPV perpetration was 4% lower compared to those headed by men (aPR = 0.96; 95% CI: 0.91–1.01). Generally, these results were consistent in the region-stratified analysis; though in Western Africa, men in female-headed households were less likely to perpetrate IPV, and in Central Africa earning disparity was not associated with IPV perpetration (Tables D-G in S1 Text).

### Association of past-year physical and/or sexual intimate partner violence with men’s sexual behaviors and HIV seropositivity

After adjustments, men who perpetrated IPV in the past year were 37% more likely to have paid for sex in the past year (aPR = 1.37; 95%CI: 1.25–1.51) and 26% more likely to have had two or more sexual partners in the past year (aPR = 1.26; 95%CI: 1.22–1.29). Men who perpetrated IPV were 9% (aPR = 1.09; 95%CI: 1.01–1.16) more likely to be living with HIV (Table 3).

**Table 3:** Crude and adjusted prevalence ratios of the association between past-year perpetration of physical and/or sexual intimate partner violence and behavioral risk factors for HIV acquisition, and HIV seropositivity among male partners.

Outcome	N_survey_	N_ind_	Crude prevalence ratio (95% CI)	Adjusted prevalence ratio (95% CI)
**Man living with HIV** ^**§**^	31	53,613		
Yes			0.94 (0.89, 1)	1.09 (1.01, 1.16)
No			Referent	Referent
**Male reported condom use at last sex with the most recent partner in past 12 months** ^¥^	48	106,452	1.00 (0.96, 1.05)	1.04 (0.99, 1.09)
Yes			Referent	Referent
No				
**Male reported number of sex partners in the past 12 months** ^¥^	47	107,909		
2+			1.23 (1.2, 1.27)	1.26 (1.22, 1.29)
1			Referent	Referent
**Male reported point-prevalence of concurrency** ^*^ ^¥^	47	19,066		
Yes			0.89 (0.86, 0.92)	1.01 (0.97, 1.04)
No			Referent	Referent
**Male reported payment for sex in the past 12 months** ^¥^	43	101,460		
Yes			1.38 (1.26, 1.52)	1.37 (1.25, 1.51)
No			Referent	Referent

^¥^Adjusted for male age (five-year age groups), wealth quintile, male education (none, primary, secondary, higher), residence type (rural, urban), survey identifier.

^**§**^Adjusted for male age (five-year age groups), wealth quintile, male education (none, primary, secondary, higher), residence (rural, urban), survey identifier, men’s lifetime number of sex partners (1, 2, ≥3).

N_ind_ = Number of individuals in adjusted analyses; N_survey_ = Number of surveys in adjusted analyses.

*Denominator includes men who had two or more sexual partners in the past year.

Heterogeneity of the crude PRs across surveys were small for concurrency (I^2^ = 8%; Fig E in S1 Text) and HIV status (I^2^ = 15%; Fig D in S1 Text) moderate for payment for sex (I^2^ = 27%; Fig B in S1 Text) and men’s past year condom use (I^2^ = 43%; Fig A in S1 Text); and high for number of sex partners (I^2^ = 66%; Fig C in S1 Text). In sensitivity analyses we found that survey region and survey year combined explained 78% and 79% of the heterogeneity across studies for the number of sex partners and payment for sex, respectively. Survey regions alone accounted for 67% of heterogeneity for past year condom use.

Our sensitivity analysis (Text B in S1 Text) did not indicate a notable impact of selection bias on the association between past year perpetration of IPV and male HIV seroprevalence.

### The role of male-perpetrated physical and/or sexual intimate partner violence in adolescent girls and young women’s risk of HIV seroprevalence

Among AGYW living with HIV, 50% (N_ind_ = 435/873) of male partners were also HIV seropositive. Crudely, this proportion did not vary by IPV perpetration status. 49% percent (N_ind_ = 128/261) of male IPV perpetrators were living with HIV, compared to 50% (N_ind_ = 300/599) to non-perpetrators. (Table H in S1 Text).

**Fig 2:**
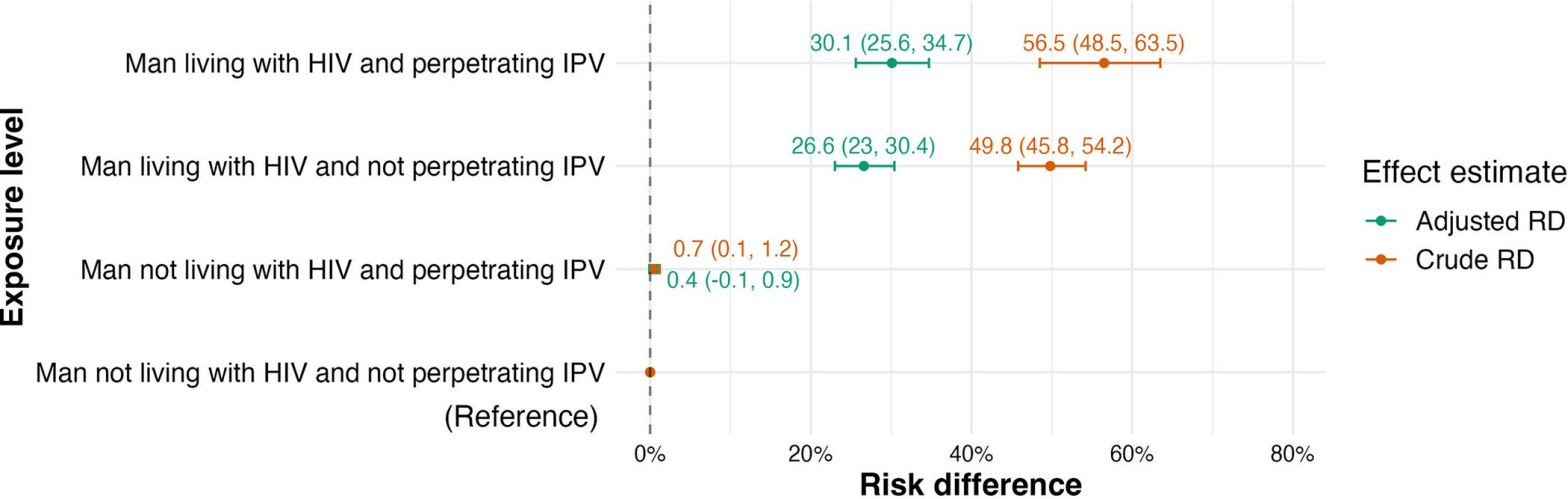
Unique and joint contributions of male partner HIV status and male partner perpetrated physical and/or sexual intimate partner violence to HIV status among adolescent girls and young women. We present crude and adjusted risk differences. Adjusted for women’s age (continuous), wealth quintile, women’s education (none, primary, secondary, higher), residence type (rural, urban), women’s lifetime number of sexual partners (1, 2, ≥3), survey identifier. IPV = Intimate Partner Violence; RD = Risk Difference.

Compared to AGYW whose partner was not living with HIV and was not perpetrating IPV, our adjusted effect modification analysis suggests AGYW whose partner was living with HIV had a 26.6% higher risk of being HIV seropositive on an absolute scale (aRD = 26.6%; 95%CI: 23.0–30.4%). Compared to the same reference group, AGYW whose male partner perpetrated IPV *in addition to living with HIV* had a 30.1% higher risk of HIV seropositivity (aRD = 30.1%; 95%CI: 25.6–34.7%; Fig 2, Tables I and J, Text A in S1 Text).

The results from the EMM analyses suggest that the *expected* joint effect of male HIV status and IPV perpetration on AGYW’s HIV status was 27 excess women living with HIV per 100. That is, the sum of the unique effects of IPV (0.4%) and male HIV status (26.6%) (Fig 2). However, the *observed* joint effect (30.1%) indicates that HIV risk in AGYW whose IPV perpetrating partner lives with HIV exceeds by 3 cases per 100 women the *expected* joint effect (27.0%) of male HIV status and IPV. This is derived as the difference between expected and observed effects: 30.1% minus 27.0%, and indicates a small additive effect measure modification between IPV perpetration and male HIV seropositivity among AGYW. The RERI was 1.03 (95% CI: 0.99–1.08) indicating an additive EMM. When performing this analysis on all women 15 years or older, we found no added effect of IPV (Table K and Text A in S1 Text). Further details on these calculations are available in Text A in S1 Text.

Among men living with HIV, a lower proportion of male perpetrators of IPV were on ART (47%; N_ind_ = 52/111) and virally suppressed (42%; N_ind_ = 47/111) compared to those who were non-perpetrators (65% for ART [N_ind_ = 996/1,541] and 60% [N_ind_ = 919/1,541] for viral suppression, respectively). However, the adjusted analyses of IPV’s effects on ART uptake (cPR = 0.73; 95%CI: 0.60–0.89; aPR = 0.88; 95%CI: 0.72–1.07; N_ind_ = 1,595) and viral load suppression (cPR = 0.73; 95%CI: 0.58–0.90; aPR = 0.84; 95%CI: 0.68–1.04; N_ind_ = 1,594) were imprecise due to the small sample size.

Sensitivity analyses showed that the removal of women who had two or more sex partners in the past year from our analyses did not change our estimates of the contribution of IPV in AGYW’s risk of HIV seroprevalence (Table L and Text A in S1 Text).

## Discussion

Pooling data on up to 111,600 couples from 48 surveys from 27 countries in Africa, we found that men whose partners reported that they perpetrate IPV were more likely to share behaviors that increased men’s risk of HIV acquisition and transmission than those who do not. These men were also more likely to be living with HIV. Further, AGYW whose male partners perpetrated IPV had a small (3%) added risk of living with HIV in addition to the risk entailed solely by their partners’ HIV status.

A few factors could explain the small additional risk of living with HIV arising from IPV. First, IPV perpetrators may be less likely to be in HIV care than non-perpetrators and have unsuppressed viral load which can increase HIV transmission risk to their female partners. Previous work has shown that unsuppressed viral load, though not ART interruptions, are more frequent among men who perpetrate IPV in crude analyses [36]. This is aligned with our study, though our sample size was insufficient to precisely estimate the association between men’s engagement in care and IPV. Second, IPV could have adverse mental health effects on women which can influence subsequent sexual behaviors, such as concurrency, substance use during sex and participation in transactional sex [14]. Our crude analysis found that among all women who have experienced past-year IPV, more had concurrent sex partners (though we did not explore the directionality of this relationship). Third, type of sex act, information which was not available in our surveys, could explain the IPV-HIV relationship among women. Coerced sex may lead to frequent anal intercourse [37, 38], often more common in IPV perpetrators [13], and mucosal lesions which increase women’s risk of HIV acquisition [39]. Among all women, IPV did not add to the risk of HIV seropositivity beyond the one resulting from male partners’ HIV status. This could be explained by the overall lower HIV incidence in older women and declining prevalence of past year IPV with age [1].

Our results align with previous research showing that IPV perpetrators are more likely to engage in sexual behaviors that increase their HIV acquisition risk and may be more likely to be living with HIV [19, 40]. Previous work has suggested that IPV perpetration and behaviors increasing men’s HIV risk could have a common root in a unifying ideal of masculinity which emphasizes heterosexual performance and dominance over women [41, 42]. The latter is compatible with our analysis of the correlates of IPV perpetration, where we show that variables reflecting women’s power within the relationship are correlated with IPV perpetration. Women who have a decision-making capacity in the household are less likely to experience IPV. However, women who earn more than their partner were more likely to experience IPV in our sample. Relative resource theory posits that when women are socioeconomically favored compared to their partner, they are at a higher risk of IPV as this goes against the traditional gender norms and can be perceived to threaten the male role [43–45]. Also, economic empowerment has been previously linked with increased sexual autonomy in women, including the ability to refuse sex, or to negotiate condom use while having sex [46, 47]. While more sexual agency would reduce women’s HIV risk, it might prompt further IPV, as shown in previous work [48, 49].

Finally, male accepting attitudes on IPV as well as frequent alcohol use were also correlated with IPV perpetration [50]. These factors diminish women’s ability to control the timing of and circumstances around sex, especially during adolescence and youth, which could contribute to the spike in the risk of HIV acquisition among AGYW, as suggested by our results. Our findings align with previous work suggesting men’s rationalization of IPV may increase the risk of IPV perpetration, and is linked with gender norms around masculinity and female subordination [51].

Our results should be interpreted considering their limitations. First, we assumed that the male partners of women who reported past-year IPV and are currently in a partnership, were the perpetrators of IPV. This is especially relevant given the reported discordance between cohabiting couple’s reports of violence [52]. Due to the challenges of gathering accurate information from men about their enactment of this type of violence [53], we believe this is the most accurate measure of IPV perpetration. Previous studies have also used this method to identify perpetrators of IPV [13, 54]. Further, while both men and women might underreport IPV, men tend to underreport both victimization and perpetration more frequently compared to women [52, 55]. Finally, only 0.7% of women in our sample had two or more sex partners in the past year and the removal of this group in the sensitivity analyses did not change our results. Second, we used HIV seropositivity data which makes it difficult to identify the direction and timing of HIV acquisition/transmission in the analysis of male HIV seropositivity. It remains possible that men acquired HIV from their female partners as opposed to outside this relationship, which could subsequently result in IPV. However, since men’s sexual behaviors pointed towards their higher HIV acquisition risk among IPV perpetrators compared to non-perpetrators, this is less likely. Third, in our analysis that uncovers IPV’s role as an effect modifier, IPV could have taken place after women’s HIV acquisition. This risk was reduced by restricting our analysis to younger women. Still, bias remains possible since we are not able to precisely disentangle the temporality between IPV and HIV. Similarly, the relationship between the IPV and male behaviors could be bidirectional; for example, alcohol consumption has been found to be associated with IPV [50]. However, our analysis is restricted to male sexual behaviors which are more likely to be subsequent to IPV given the underlying gender attitudes. Fourth, IPV and sexual behaviors were self-reported and might be subject to under-reporting due to their sensitive nature, which could dilute the association between the two. However, the surveys took measures to ensure confidentiality; for example DHS does not administer the survey unless complete privacy is achieved [56, 57]. Additionally only one participant per household (per fraction of households in some DHS surveys) was selected for the domestic violence module such that other household members were not aware of what was being discussed during the interview [56, 57]. Fifth, heterogeneity of effect size measures across surveys in univariate analyses was sometimes moderate to high, depending on the outcome. However, controlling for survey-level fixed effects helps account for measured and unmeasured differences by country and survey year and our subgroup analyses suggest that region and survey year accounted for a notable part of this heterogeneity.

Our study also has several strengths. First, we conducted a comprehensive analysis of the HIV status of male partners, their engagement in HIV care and sexual behaviors to elucidate the pathways between IPV and women’s HIV acquisition. Second, our large sample size of cohabiting male-female dyads from population-based surveys, which includes detailed information on more than 111,600 couples, allowed us to estimate IPV’s added effect on women’s absolute risk of living with HIV. Finally, we conducted a multitude of sensitivity analyses to ensure the robustness of our results.

Ending IPV may not single-handedly eliminate HIV acquisition in women since the added risk of living with HIV due to IPV, beyond the risk entailed solely in their partners’ HIV status could be small. Still, experiencing IPV adds to AGYW’s risk of living with HIV, which demonstrates the mutually reinforcing effects of HIV/IPV and the importance of addressing both issues simultaneously. Women’s empowerment-based HIV/IPV prevention interventions are crucial and should focus on AGYW, who are at the highest risk of both IPV and HIV acquisition [58]. The UNGA Political Declaration on HIV/AIDS commits to the delivery of integrated services for HIV prevention, focused on strengthening economic independence, sexual agency and challenging gender stereotypes [58]. Meaningful involvement of both men and women in the development of these multi-pronged services tailored to the needs of women of all ages is important to develop effective approaches for IPV prevention [59]. Community-based efforts that foster women’s agency and combat negative social norms in men may be key in dislodging the well‐established gender inequalities driving both IPV and HIV [59]. However, gender-based discrimination in social norms, practices and laws varies widely across the countries included in our study [60]. These local and regional variations should be accounted for in the development of services for HIV and IPV prevention. So far, existing population-based surveys have understandably focused on women and their reported experience of IPV. Despite the methodological challenges with gathering information from and about men on their own use of violence, collecting data on IPV perpetrators is crucial to devise methods for IPV and HIV prevention in women. Longitudinal studies are needed to further disentangle causal pathways between male-perpetrated IPV and HIV acquisition in women, to subsequently inform IPV and HIV prevention interventions. Violence beyond IPV, such as non-partner sexual violence and violence among transactional relationships are equally concerning and have implications for HIV acquisition risk. The impacts of violence and HIV are profound and have long-lasting effects on the well-being of millions of women and girls globally. Actions to eliminate violence and end AIDS must be accelerated.

## Supporting information

S1 Text **Table A.** Operational definitions of past year physical and/or sexual intimate partner violence and indicators most frequently used in surveys included in this analysis. **Table B.** The summary of exposures, outcomes and covariates included in the analyses of each of the three research questions. **Table C.** Distribution of past year physical and/or sexual intimate partner violence (IPV) stratified by surveys and regions. **Table D.** Crude and adjusted prevalence ratios of the association between partnership and male individual characteristics and perpetration of past year physical and/or sexual intimate partner violence in Central Africa. **Table E.** Crude and adjusted prevalence ratios of the association between partnership and male individual characteristics and perpetration of past year physical and/or sexual intimate partner violence in Western Africa. **Table F.** Crude and adjusted prevalence ratios of the association between partnership and male individual characteristics and perpetration of past year physical and/or sexual intimate partner violence in Eastern Africa. **Table G.** Crude and adjusted prevalence ratios of the association between partnership and male individual characteristics and perpetration of past year physical and/or sexual intimate partner violence in Southern Africa. **Table H.** HIV seroprevalence among male partners of adolescent girls and young women living with HIV. The proportions are stratified by perpetration/experience of physical and/or sexual intimate partner violence in the past year. **Fig A.** Survey-specific and pooled crude prevalence ratios (PR) for past year condom use at last sex with the most recent partner among men who had perpetrated past year physical and/or sexual intimate partner violence (IPV) compared to men who had not. **Fig B.** Survey-specific and pooled crude prevalence ratios (PR) for past year payment for sex among men who had perpetrated past year physical and/or sexual intimate partner violence (IPV) compared to men who had not. **Fig C.** Survey-specific and pooled crude prevalence ratios (PR) for two or more sex partners in the past year among men who had perpetrated past year physical and/or sexual intimate partner violence (IPV) compared to men who had not. **Fig D.** Survey-specific and pooled crude prevalence ratios (PR) for HIV prevalence among men who had perpetrated past year physical and/or sexual intimate partner violence (IPV) compared to men who had not. **Fig E.** Survey-specific and pooled crude prevalence ratios (PR) for concurrency among men who had perpetrated past year physical and/or sexual intimate partner violence (IPV) compared to men who had not. **Text A.** Detailed methodology and the results for the analysis of the role of male-perpetrated physical and/or sexual intimate partner violence in women’s risk of HIV seroprevalence. **Table I, Text A.** Crude and adjusted absolute risks of living with HIV among AGYW who have HIV seropositive male partner perpetrating IPV, who have HIV seropositive partner not perpetrating IPV, who have HIV seronegative partner perpetrating IPV, and who have HIV seronegative partner not perpetrating IPV. **Table J, Text A.** Unique and joint contributions of male partner HIV status and male partner perpetrated physical and/or sexual IPV to HIV status among adolescent girls and young women. We present crude and adjusted risk differences. **Table K, Text A.** Unique and joint contributions of male partner HIV status and male partner perpetrated IPV to female HIV status among all women over the age of 15. We present crude and adjusted risk differences. **Table L, Text A.** Unique and joint contributions of male partner HIV status and male partner perpetrated physical and/or sexual IPV to HIV status among adolescent girls and young women, excluding women who had two or more sexual partners in the past year. We present crude and adjusted risk differences. **Text B.** Sensitivity analysis for the effects of selection bias on male HIV seroprevalence analysis. **Table M, Text B.** Bias parameter values and data sources used for the selection bias sensitivity analysis. **Table N, Text B.** Effect of various bias parameter values on corrected prevalence ratio. **Text C.** STROBE Statement.
(DOCX)
