# Supplementary material for: Characteristics of male perpetrators of intimate partner violence and implications for women’s HIV status: A pooled analysis of cohabiting couples from 27 countries in Africa (2000–2020)
Source: PLOS Glob Public Health. 2023 Sep 6;3(9):e0002146. doi: 10.1371/journal.pgph.0002146 (PMC10482294; doi:10.1371/journal.pgph.0002146)
Supplement: S1 Text — Table A. Operational definitions of past year physical and/or sexual intimate partner violence and indicators most frequently used in surveys included in this analysis. Table B. The summary of exposures, outcomes and covariates included in the analyses of each of the three research questions. Table C. Distribution of past year physical and/or sexual intimate partner violence (IPV) stratified by surveys and regions. Table D. Crude and adjusted prevalence ratios of the association between partnership and male individual characteristics and perpetration of past year physical and/or sexual intimate partner violence in Central Africa. Table E. Crude and adjusted prevalence ratios of the association between partnership and male individual characteristics and perpetration of past year physical and/or sexual intimate partner violence in Western Africa. Table F. Crude and adjusted prevalence ratios of the association between partnership and male individual characteristics and perpetration of past year physical and/or sexual intimate partner violence in Eastern Africa. Table G. Crude and adjusted prevalence ratios of the association between partnership and male individual characteristics and perpetration of past year physical and/or sexual intimate partner violence in Southern Africa. Table H. HIV seroprevalence among male partners of adolescent girls and young women living with HIV. The proportions are stratified by perpetration/experience of physical and/or sexual intimate partner violence in the past year. Fig A. Survey-specific and pooled crude prevalence ratios (PR) for past year condom use at last sex with the most recent partner among men who had perpetrated past year physical and/or sexual intimate partner violence (IPV) compared to men who had not. Fig B. Survey-specific and pooled crude prevalence ratios (PR) for past year payment for sex among men who had perpetrated past year physical and/or sexual intimate partner violence (IPV) compared to men who had not. Fig C [file pgph.0002146.s001.docx]

**S1 Text - Supporting Information**

**Table A in S1 Text.** Operational definitions of past year physical and/or sexual intimate partner violence and indicators most frequently used in surveys included in this analysis.

|  | **Questions used to define the measures in DHS**^¥§^ | **Questions used to define the measures in PHIA** | |
| --- | --- | --- | --- |
| **Past year physical and/or sexual IPV** | Did your (last) (husband/partner) ever do any of the following things to you?  If the respondent answers “Yes”:  How often did this happen during the last 12 months: often, only sometimes, or not at all?  *Physical violence*   - Push you, shake you, or throw something at you? - Slap you? - Punch you with his fist or with something that could hurt you? - Kick you, drag you, or beat you up? - Choke you or burn you on purpose ? - Threaten or attack you with a knife, gun, or other weapon?   *Sexual violence*   - Physically force you to have sexual intercourse with him when you did not want to? - Physically force you to perform any other sexual acts you did not want to? - Force you with threats or in any other way to perform sexual acts you did not want to? | | In the past 12 months, did a partner do any of these things to you? By partner, I mean a life-in partner, whether or not you were married at the time.  *Physical violence*   - Slapped you, threw something at you that could hurt you, pushed you or shoved you? - Punched, kicked, whipped, or beat you with an object? - Choked smothered, tried to drown you, or burned you intentionally? - Used or threatened you with a knife, gun or other weapon?   *Sexual violence*   - In the past 12 months, did a partner physically force you to have sex? - In the past 12 months, did a partner pressure you to have sex and did succeed? |

§ Question “(Does/did) your (last) husband/partner ever twist your arm or pull your hair?” was removed from the definition of past year physical IPV to align the DHS and PHIA definitions.

¥ In N_s_= 9 DHS surveys “threatening to attack” and “attack” questions were asked as two separate questions.

*DHS=Demographic and Health Survey; IPV=intimate partner violence; PHIA=Population-based HIV Impact Assessment Survey.*

**Table B in S1 Text.** The summary of exposures, outcomes and covariates included in the analyses of each of the three research questions.

| **Research question** | **Exposure(s)** | **Outcome** | **Adjustment variables** |
| --- | --- | --- | --- |
| a) What male partner and partnership-level characteristics are associated with IPV? | Individual   - Male accepting attitudes on IPV - Man has more than one wife/cohabiting partner - Male alcohol use frequency   Partnership   - Couple age disparity - Couple earning disparity - Women has a say in household decision-making - Household headship (male/female) | Perpetration of physical and/or sexual IPV in the past 12 months | Male age (five-year age group), household wealth quintile, male education (none, primary, secondary, higher) residence type (rural, urban), survey identifier. |
| b) Are men who are reported to perpetrate IPV more likely to report behaviors that increase their risk of HIV acquisition and to be living with HIV? | Perpetration of physical and/or sexual IPV in the past 12 months | Condom use at last sex with the most recent partner in the last 12 months | Male age (five-year age group), household wealth quintile, male education (none, primary, secondary, higher) residence type (rural, urban), and survey identifier |
|  |  | Number of sex partners in the past 12 months |  |
|  |  | Male reported point-prevalence of concurrency (having more than one sexual partnership at a single point in time six months before the interview) |  |
|  |  | Payment for sex in the past 12 months |  |
|  |  | Male HIV status | Male age (five-year age group), household wealth quintile, male education (none, primary, secondary, higher) residence type (rural, urban), men’s lifetime sex partners (1, 2, $\geq3$), survey identifier. |
| c) Does experiencing IPV increase young women’s risk of living with HIV, beyond the risk entailed by their male partner’s HIV status? | Male HIV status | Female HIV status | Female age (five-year age group or continuous), household wealth quintile, female education (none, primary, secondary, higher) residence type (rural, urban), women’s lifetime sex partners (1, 2, $\geq3$), survey identifier, and a product term between male HIV status and past year IPV perpetration |

*ART= antiretroviral treatment; IPV = intimate partner violence; VLS= viral load suppression; ELISA = enzyme-linked immunosorbent assay.*

###

**Table C in S1 Text.** Distribution of past year physical and/or sexual intimate partner violence (IPV) stratified by surveys and regions.

| **Country** | **Survey year** | **Survey type** | **Total sample size** | **Past year physical and/or sexual IPV, N (%)** |
| --- | --- | --- | --- | --- |
| **Overall** |  |  | 111,659 | 23,777 (21.3) |
| **Central Africa** | |  |  |  |
| Angola | 2015 | DHS | 2,034 | 532 (26.2%) |
| Cameroon | 2018 | DHS | 904 | 207 (22.9%) |
| Gabon | 2012 | DHS | 1,513 | 512 (33.8%) |
| Sao Tome and Principe | 2008 | DHS | 823 | 250 (30.4%) |
| Tchad | 2014 | DHS | 2,350 | 375 (16.0%) |
| **Total** |  |  | 7,624 | 1,876 (24.6) |
| **Western Africa** | |  |  |  |
| Burkina Faso | 2010 | DHS | 3,728 | 388 (10.4%) |
| Cote d'Ivoire | 2012 | DHS | 1,574 | 352 (22.4%) |
| Ghana | 2008 | DHS | 1,042 | 194 (18.6%) |
| Gambia | 2013 | DHS | 762 | 76 (10.0%) |
| Gambia | 2019 | DHS | 901 | 133 (14.8%) |
| Liberia | 2007 | DHS | 2,235 | 816 (36.5%) |
| Liberia | 2019 | DHS | 1,436 | 530 (36.9%) |
| Mali | 2006 | DHS | 1,935 | 302 (15.6%) |
| Mali | 2012 | DHS | 2,059 | 566 (27.5%) |
| Mali | 2018 | DHS | 2,337 | 479 (20.5%) |
| Nigeria | 2008 | DHS | 6,751 | 966 (14.3%) |
| Nigeria | 2013 | DHS | 6,961 | 778 (11.2%) |
| Nigeria | 2018 | DHS | 6,386 | 947 (14.8%) |
| Sierra Leone | 2019 | DHS | 2,652 | 1,026 (38.7%) |
| Togo | 2013 | DHS | 1,752 | 265 (15.1%) |
| **Total** |  |  | 42,511 | 7,818 (18.4) |
| **Eastern Africa** | |  |  |  |
| Burundi | 2016 | DHS | 1,481 | 481 (32.5%) |
| Ethiopia | 2016 | DHS | 2,687 | 463 (17.2%) |
| Kenya | 2003 | DHS | 1,190 | 336 (28.2%) |
| Kenya | 2008 | DHS | 1,251 | 403 (32.2%) |
| Kenya | 2014 | DHS | 2,283 | 582 (25.5%) |
| Comoros | 2012 | DHS | 594 | 24 (4.0%) |
| Malawi | 2004 | DHS | 1,709 | 336 (19.7%) |
| Malawi | 2010 | DHS | 3,365 | 740 (22.0%) |
| Malawi | 2015 | DHS | 3,379 | 823 (24.4%) |
| Malawi | 2015 | PHIA | 3,300 | 166 (5.0%) |
| Mozambique | 2015 | AIS | 1,158 | 170 (14.7%) |
| Rwanda | 2005 | DHS | 1,887 | 425 (22.5%) |
| Rwanda | 2010 | DHS | 2,450 | 1,219 (49.8%) |
| Rwanda | 2015 | DHS | 1,377 | 306 (22.2%) |
| Rwanda | 2019 | DHS | 1,387 | 350 (25.2%) |
| Tanzania | 2010 | DHS | 978 | 296 (30.3%) |
| Tanzania | 2015 | DHS | 1,278 | 386 (30.2%) |
| Uganda | 2016 | PHIA | 613 | 78 (12.7%) |
| Zambia | 2007 | DHS | 2,689 | 1,113 (41.4%) |
| Zambia^*^ | 2013 | DHS | 6,095 | 1,748 (28.7%) |
| Zambia | 2016 | PHIA | 3,205 | 122 (3.8%) |
| Zambia | 2018 | DHS | 4,629 | 1,203 (26%) |
| Zambia | 2005 | DHS | 2,132 | 709 (33.3%) |
| Zimbabwe | 2010 | DHS | 2,479 | 731 (29.5%) |
| Zimbabwe | 2015 | DHS | 2,966 | 638 (21.5%) |
| Zimbabwe | 2015 | PHIA | 3,543 | 154 (4.3%) |
| **Total** |  |  | 60,105 | 14,002 (23.3%) |
| **Southern Africa** | |  |  |  |
| Eswatini | 2016 | PHIA | 924 | 28 (3.0%) |
| South Africa | 2016 | DHS | 495 | 53 (10.7%) |
| **Total** |  |  | 1,419 | 81 (5.7%) |

*DHS = Demographic and Health Surveys; IPV=intimate partner violence; PHIA = Population-based HIV Impact Assessment Survey.*

*Removed from the HIV seroprevalence analyses.

**Table D in S1 Text.** Crude and adjusted prevalence ratios of the association between partnership and male individual characteristics and perpetration of past year physical and/or sexual intimate partner violence in Central Africa.

| **Partnership characteristics** | **N_survey_** | **N_ind_** | **Crude prevalence ratio (95%CI)** | **Adjusted prevalence ratio**  **(95%CI)^†^** |
| --- | --- | --- | --- | --- |
| **Couple earning disparity** | 5 | 7,215 |  |  |
| Less than him |  |  | Referent | Referent |
| Same |  |  | 0.88 (0.71, 1.08) | 0.86 (0.7, 1.05) |
| More than him |  |  | 1.12 (0.91, 1.38) | 1.08 (0.88, 1.31) |
| Woman not paid in cash/kind |  |  | 0.92 (0.84, 1.01) | 0.93 (0.85, 1.02) |
| **Mean couple age disparity** | 5 | 7,573 | 0.98 (0.97, 0.99) | 0.99 (0.98, 1.00) |
| **Woman has a say in household decision-making** | 5 | 7,551 |  |  |
| Yes |  |  | 0.93 (0.85, 1.02) | 0.86 (0.78, 0.94) |
| No |  |  | Referent | Referent |
| **Household head** | 5 | 7,573 |  |  |
| Female |  |  | 1.08 (0.91, 1.29) | 1.01 (0.85, 1.19) |
| Male |  |  | Referent | Referent |
| **Male individual characteristics** |  |  |  |  |
| **Male accepting attitudes on IPV** | 5 | 7,435 |  |  |
| Yes |  |  | 1.24 (1.13, 1.35) | 1.26 (1.15, 1.38) |
| No |  |  | Referent | Referent |
| **Man has more than one wife/cohabiting partner** | 5 | 7,573 |  |  |
| Yes |  |  | 0.98 (0.89, 1.09) | 1.18 (1.06, 1.32) |
| No |  |  | Referent | Referent |
| **Male alcohol use frequency** |  |  |  |  |
| Never | 5 | 7,558 | Referent | Referent |
| Sometimes |  |  | 2.06 (1.85, 2.28) | 1.92 (1.74, 2.13) |
| Often |  |  | 3.22 (2.93, 3.54) | 3.13 (2.84, 3.45) |

*IPV= intimate partner violence; N_ind_ = Number of individuals in the adjusted analyses; N_survey_ = Number of surveys in the adjusted analyses.*

† All models are adjusted for male age (five-year age groups), male education (none, primary, secondary, higher) wealth quantile, residence type (rural, urban), survey identifier.

**Table E in S1 Text.** Crude and adjusted prevalence ratios of the association between partnership and male individual characteristics and perpetration of past year physical and/or sexual intimate partner violence in Western Africa.

| **Partnership characteristics** | **N_survey_** | **N_ind_** | **Crude prevalence ratio (95%CI)** | **Adjusted prevalence ratio (95%CI)^†^** |
| --- | --- | --- | --- | --- |
| **Couple earning disparity** | 15 | 40,347 |  |  |
| Less than him |  |  | Referent | Referent |
| Same |  |  | 0.94 (0.84, 1.06) | 0.86 (0.77, 0.96) |
| More than him |  |  | 1.19 (1.06, 1.33) | 1.14 (1.02, 1.27) |
| Woman not paid in cash/kind |  |  | 1.05 (1.01, 1.10) | 0.95 (0.91, 1.00) |
| **Mean couple age disparity** | 15 | 42,289 | 0.99 (0.99, 1.00) | 1.00 (1.00, 1.01) |
| **Woman has a say in household decision-making** | 15 | 42,229 |  |  |
| Yes |  |  | 0.94 (0.89, 0.98) | 0.86 (0.82, 0.90) |
| No |  |  | Referent | Referent |
| **Household head** | 15 | 42,289 |  |  |
| Female |  |  | 1.09 (0.97, 1.23) | 0.89 (0.80, 0.98) |
| Male |  |  | Referent | Referent |
| **Male individual characteristics** |  |  |  |  |
| **Male accepting attitudes on IPV** | 14 | 39,886 |  |  |
| Yes |  |  | 1.25 (1.20, 1.31) | 1.21 (1.16, 1.26) |
| No |  |  | Referent | Referent |
| **Man has more than one wife/cohabiting partner** | 15 | 42,260 |  |  |
| Yes |  |  | 1.01 (0.97, 1.06) | 1.10 (1.04, 1.16) |
| No |  |  | Referent | Referent |
| **Male alcohol use frequency** |  |  |  |  |
| Never | 15 | 42,221 | Referent | Referent |
| Sometimes |  |  | 2.02 (1.92, 2.13) | 2.0 (1.90, 2.11) |
| Often |  |  | 2.97 (2.79, 3.16) | 2.96 (2.78, 3.16) |

*IPV= intimate partner violence; N_ind_ = Number of individuals in the adjusted analyses; N_survey_ = Number of surveys in the adjusted analyses.*

† All models are adjusted for male age (five-year age groups), male education (none, primary, secondary, higher) wealth quantile, residence type (rural, urban), survey identifier.

**Table F in S1 Text**. Crude and adjusted prevalence ratios of the association between partnership and male individual characteristics and perpetration of past year physical and/or sexual intimate partner violence in Eastern Africa.

| **Partnership characteristics** | **N_survey_** | **N_ind_** | **Crude prevalence ratio**  **(95%CI)** | **Adjusted prevalence ratio (95%CI)^†^** |
| --- | --- | --- | --- | --- |
| **Couple earning disparity** | 22 | 47,023 |  |  |
| Less than him |  |  | Referent | Referent |
| Same |  |  | 0.91 (0.85, 0.97) | 0.88 (0.83, 0.94) |
| More than him |  |  | 1.02 (0.95, 1.10) | 1.10 (1.02, 1.18) |
| Woman not paid in cash/kind |  |  | 0.87 (0.84, 0.91) | 0.88 (0.85, 0.91) |
| **Mean couple age disparity** | 26 | 59,486 | 0.99 (0.98, 0.99) | 1.00 (0.99, 1.00) |
| **Woman has a say in household decision-making** | 26 | 59,459 |  |  |
| Yes |  |  | 0.68 (0.66, 0.71) | 0.79 (0.76, 0.81) |
| No |  |  | Referent | Referent |
| **Household head** | 26 | 59,486 |  |  |
| Female |  |  | 0.77 (0.71, 0.83) | 0.98 (0.92, 1.05) |
| Male |  |  | Referent | Referent |
| **Male individual characteristics** |  |  |  |  |
| **Male accepting attitudes on IPV** | 23 | 51,641 |  |  |
| Yes |  |  | 1.44 (1.39, 1.48) | 1.26 (1.22, 1.3) |
| No |  |  | Referent | Referent |
| **Man has more than one wife/cohabiting partner** | 26 | 59,473 |  |  |
| Yes |  |  | 1.31 (1.24, 1.38) | 1.27 (1.21, 1.33) |
| No |  |  | Referent | Referent |
| **Male alcohol use frequency** |  |  |  |  |
| Never | 24 | 54,962 | Referent | Referent |
| Sometimes |  |  | 1.71 (1.64, 1.77) | 1.65 (1.60, 1.71) |
| Often |  |  | 2.72 (2.61, 2.84) | 2.79 (2.69, 2.90) |

*IPV= intimate partner violence; N_ind_ = Number of individuals in the adjusted analyses; N_survey_ = Number of surveys in the adjusted analyses.*

† All models are adjusted for male age (five-year age groups), male education (none, primary, secondary, higher) wealth quantile, residence type (rural, urban), survey identifier.

**Table G in S1 Text.** Crude and adjusted prevalence ratios of the association between partnership and male individual characteristics and perpetration of past year physical and/or sexual intimate partner violence in Southern Africa.

| **Partnership characteristics**^¥^ | **N_survey_** | **N_ind_** | **Crude prevalence ratio**  **(95%CI)** | **Adjusted prevalence ratio (95%CI)^†^** |
| --- | --- | --- | --- | --- |
| **Mean couple age disparity** | 2 | 1,025 | 0.97 (0.92, 1.02) | 1.00 (0.95, 1.05) |
| **Woman has a say in household decision-making** | 2 | 1,025 |  |  |
| Yes |  |  | 0.69 (0.41, 1.15) | 0.66 (0.40, 1.08) |
| No |  |  | Referent | Referent |
| **Household head** | 2 | 1,025 |  |  |
| Female |  |  | 1.08 (0.59, 1.96) | 1.25 (0.70, 2.22) |
| Male |  |  | Referent | Referent |
| **Male individual characteristics** |  |  |  |  |
| **Man has more than one wife/cohabiting partner** | 2 | 1,025 |  |  |
| Yes |  |  | 1.25 (0.63, 2.50) | 1.2 (0.58, 2.50) |
| No |  |  | Referent | Referent |
| **Male alcohol use frequency** | 2 | 1,021 |  |  |
| Never |  |  | Referent | Referent |
| Sometimes |  |  | 1.74 (1.07, 2.83) | 1.74 (1.07, 2.83) |
| Often |  |  | 2.81 (1.60, 4.94) | 2.63 (1.56, 4.42) |

*IPV= intimate partner violence; N_ind_ = Number of individuals in the adjusted analyses; N_survey_ = Number of surveys in the adjusted analyses.*

† All models are adjusted for male age (five-year age groups), male education (none, primary, secondary, higher) wealth quantile, residence type (rural, urban), survey identifier.

¥ Male accepting attitudes on IPV and couple earning disparity were not collected in Swaziland 2016 PHIA survey; to allow for adjustment by survey identifier these variables were not included in the fully adjusted models.


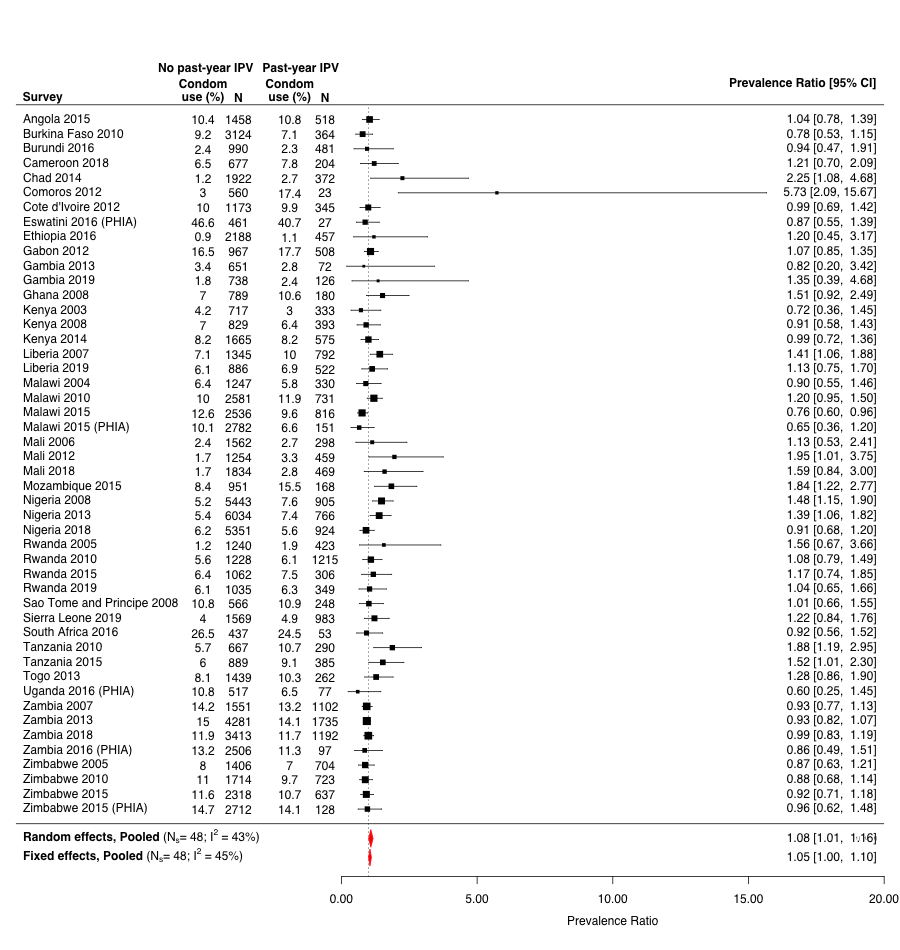


**Figure A in S1 Text.** Survey-specific and pooled crude prevalence ratios (PR) for past year condom use at last sex with the most recent partner among men who had perpetrated past year physical and/or sexual intimate partner violence (IPV) compared to men who had not. Both fixed and random effects pooled estimates are provided. After accounting for the moderating effects of survey region in the random effects analysis, I^2^= 33%.

95%CI=95% confidence intervals; IPV=intimate partner violence; N= Total number of men who had perpetrated past year IPV or did not perpetrate past year IPV (stratum-specific denominators); N_s_=number of surveys.


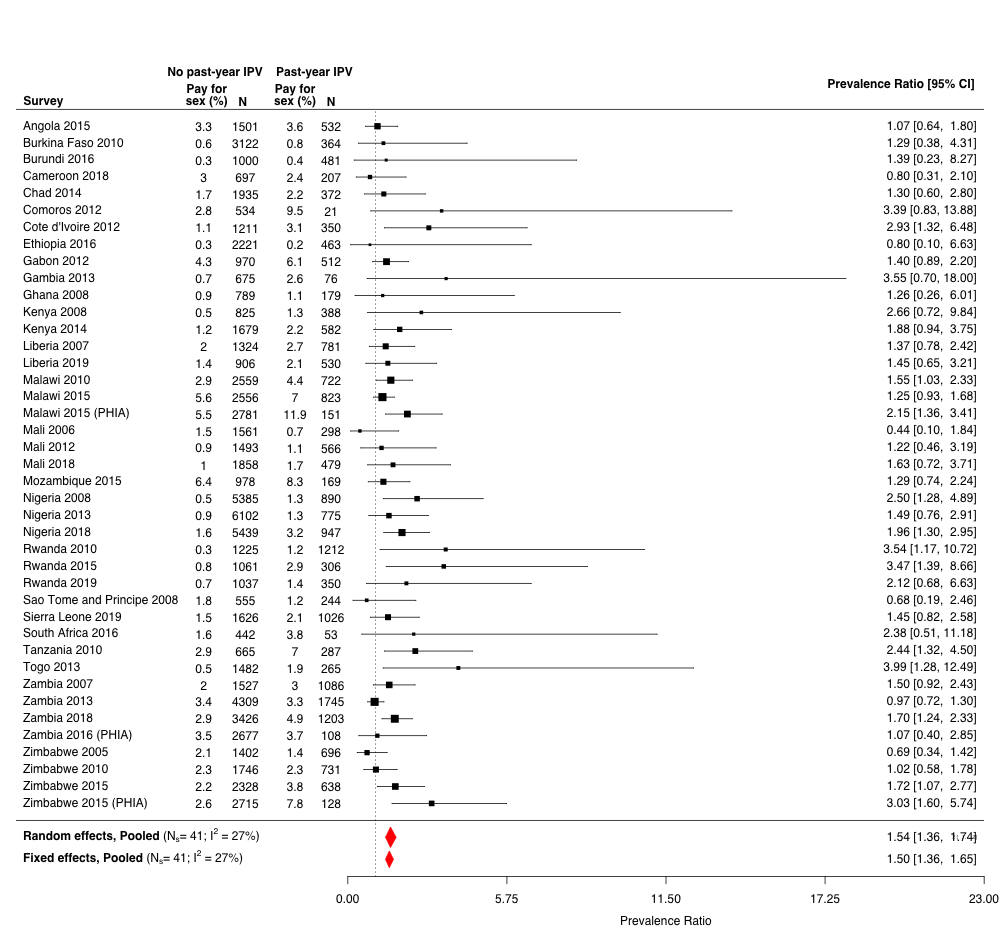


**Figure B in S1 Text.** Survey-specific and pooled crude prevalence ratios (PR) for past year payment for sex among men who had perpetrated past year physical and/or sexual intimate partner violence (IPV) compared to men who had not. Both fixed and random effects pooled estimates are provided. After accounting for the moderating effects of survey region and survey year in the random effects analysis, I^2^= 21%.

95%CI=95% confidence intervals; IPV=intimate partner violence; N= Total number of men who had perpetrated past year IPV or did not perpetrate past year IPV (stratum-specific denominators); N_s_=number of surveys.


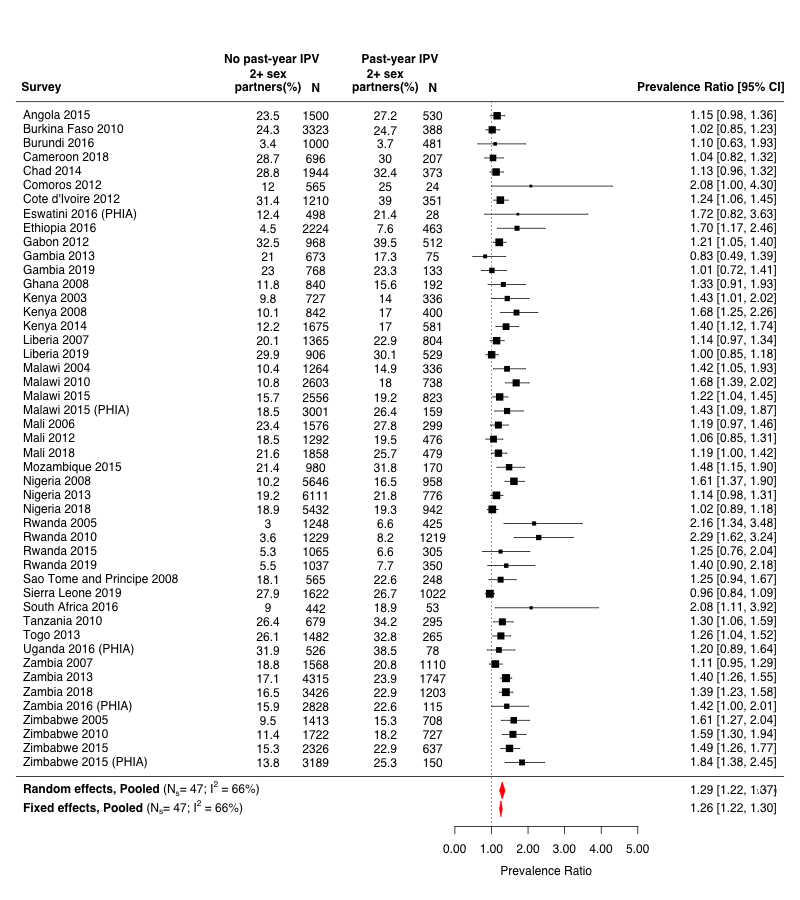


**Figure C in S1 Text.** Survey-specific and pooled crude prevalence ratios (PR) for two or more sex partners in the past year among men who had perpetrated past year physical and/or sexual intimate partner violence (IPV) compared to men who had not. Both fixed and random effects pooled estimates are provided. After accounting for the moderating effects of survey region and survey year in the random effects analysis, I^2^= 22%.

95%CI=95% confidence intervals; IPV=intimate partner violence; N= Total number of men who had perpetrated past year IPV or did not perpetrate past year IPV (stratum-specific denominators); N_s_=number of surveys.


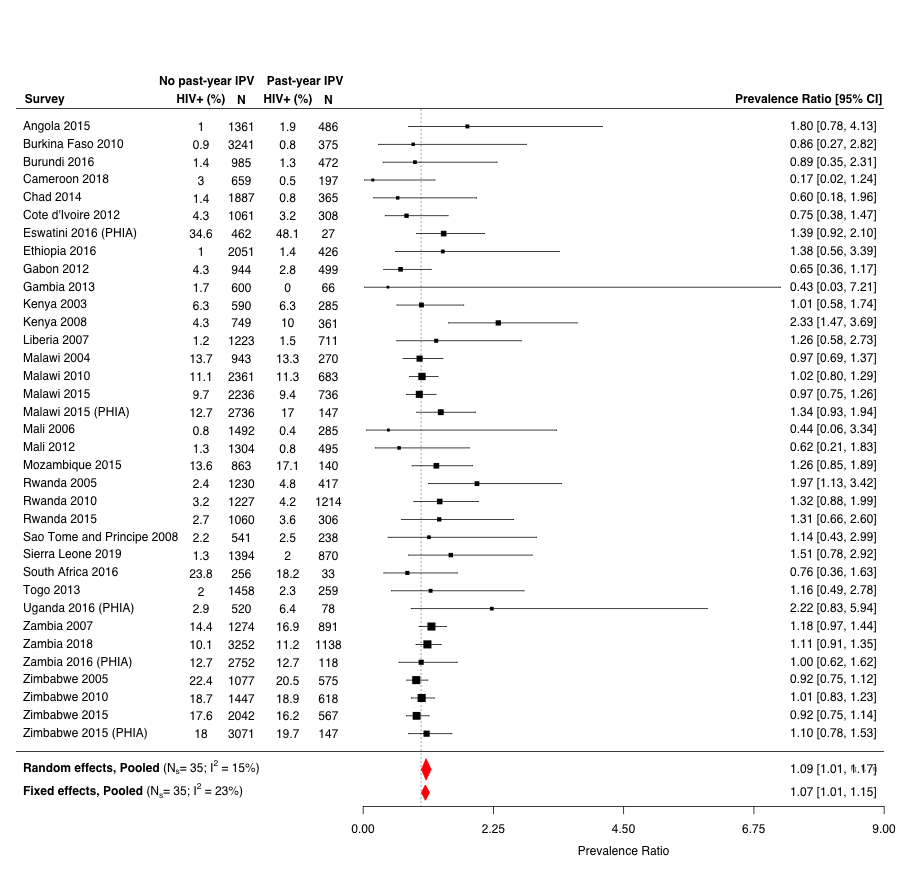


**Figure D in S1 Text.** Survey-specific and pooled crude prevalence ratios (PR) for HIV prevalence among men who had perpetrated past year physical and/or sexual intimate partner violence (IPV) compared to men who had not. Both fixed and random effects pooled estimates are provided. Zambia 2013 DHS survey has been removed from this analysis.

95%CI=95% confidence intervals; IPV=intimate partner violence; N= Total number of men who had perpetrated past year IPV or did not perpetrate past year IPV (stratum-specific denominators); N_s_=number of surveys.

**
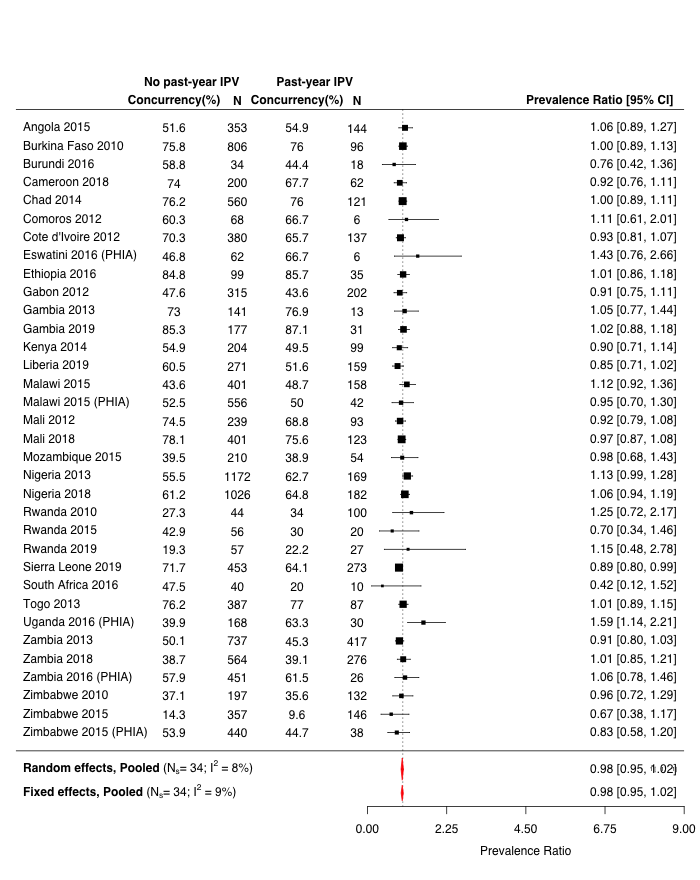
**

**Figure E in S1 Text.** Survey-specific and pooled crude prevalence ratios (PR) for concurrency among men who had perpetrated past year physical and/or sexual intimate partner violence (IPV) compared to men who had not. Both fixed and random effects pooled estimates are provided. 13 surveys were removed since no men had concurrent sexual partners.

95%CI=95% confidence intervals; IPV=intimate partner violence; N= Total number of men who had perpetrated past year IPV or did not perpetrate past year IPV (stratum-specific denominators); N_s_=number of surveys.

**Table H in S1 Text.** HIV seroprevalence among male partners of adolescent girls and young women living with HIV. The proportions are stratified by perpetration/experience of physical and/or sexual intimate partner violence in the past year.

|  | **Past year physical and/or sexual IPV, n (%)** | |  | |
| --- | --- | --- | --- | --- |
|  | **Yes**  **(N_ind_= 261)** | **No**  **(N_ind_=599)** | | **Overall^*^**  **(N_ind_= 873)** |
| Male partner HIV prevalence |  |  | |  |
| Male living with HIV | 128 (49.0 %) | 300 (50.1 %) | | 435 (49.8 %) |
| Male not living with HIV | 99 (37.9 %) | 238 (39.7 %) | | 343 (39.3 %) |
| *(Missing)* | 34 (13.0 %) | 61 (10.2 %) | | 95 (10.9 %) |

*N_ind =_ number of adolescent girls and young women living with HIV.*

*Male partner HIV prevalence among adolescent girls and young women living with HIV irrespective of male perpetration of IPV in the past year. N_ind_ = 13 women have missing data for the experience of physical and/or sexual violence in the past year.

**Text A. Detailed methodology and the results for the analysis of the role of male-perpetrated physical and/or sexual intimate partner violence in women’s risk of HIV seroprevalence**

**Methods:**

First, we calculate the absolute risk of living with HIV among adolescent girls and young women (AGYW) who a) have HIV seropositive male partner who perpetrated IPV in the past year b) have HIV seropositive male partner who did not perpetrate IPV in the past year c) have HIV seronegative male partner who perpetrated IPV in the past year. We also calculate baseline risk among women whose partner is HIV seronegative and did not perpetrate IPV in the past year. (Table I in S1 Text).

Using the baseline risk among women whose partner is HIV seronegative and does not perpetrate IPV in Table A as the reference category, we calculate the adjusted and crude risk differences (RD) for the unique and joint contributions of male partner HIV status and male partner perpetrated IPV to HIV status among AGYW (Table J in S1 Text). To calculate the risks and subsequent risk [1] differences we use marginal standardization based on GEE model with robust standard errors (Formula 1).

P( $Y_{ij}=1\left| IPV=ipv_{ij}, HIV=hiv_{ij} \right)$=$\sum_{c} P(Y_{ij}=1\left| {IPV}_{ij}={ipv}_{ij}, HIV_{ij}=hiv_{ij}, C_{ij}=c_{ij} \right)* P (C_{ij}=c_{ij}$). (Formula 1)

In Formula 1, $\boldsymbol{Y}_{\boldsymbol{ij}}$ is the marginally standardized probability of living with HIV for a woman *i* in primary sampling unit (PSU) *j,* where every observation in the population is set/fixed to have a given combination of exposure levels: [ (ipv_ij_= 1, hiv_ij_ = 1); (ipv_ij_= 1, hiv_ij_ = 0); (ipv_ij_= 0, hiv_ij_ = 1); (ipv_ij_= 0, hiv_ij_ = 0)]

$\left[ \boldsymbol{IPV=ip}\boldsymbol{v}_{\boldsymbol{ij}}\boldsymbol{, HIV=hi}\boldsymbol{v}_{\boldsymbol{ij}} \right]$reflects forcing all observations to a single combination of the above exposure levels. $\boldsymbol{C}_{\boldsymbol{ij}}\boldsymbol{=}\boldsymbol{c}_{\boldsymbol{ij}}$ refers to a combination of observed values for a confounder vector **C_ij_***.* The predicted probability of living with HIV for women *i* in PSU *j*, given each exposure combination is weighted by the relative frequency of c_ij_ and summed over each covariate pattern (combination of categorical covariates).

To calculate the estimates and associated 95% CI we used bootstrapping, where the resampling unit was the PSU.

**Results**

**Table I in S1 Text.** Crude and adjusted *absolute risks* of living with HIV among AGYW who have HIV seropositive male partner perpetrating IPV, who have HIV seropositive partner not perpetrating IPV, who have HIV seronegative partner perpetrating IPV, and who have HIV seronegative partner not perpetrating IPV.

|  | N_exp_/N_t_ | Crude risk  (95% CI) | Adjusted risk  (95% CI) | N_exp_/N_t_ | Crude risk  (95% CI) | Adjusted risk  (95% CI) |
| --- | --- | --- | --- | --- | --- | --- |
| Female HIV prevalence | **No past year IPV** | | | **Past year IPV** | | |
| Male HIV^-^ | 13,003/  17,834 | 1.9% (1.6%, 2.1%) | **1.8% (1.6%, 2.1%)** | 3,987/  17,834 | 2.6% (2.1%, 3.0%) | **2.3% (1.8%, 2.7%** |
| Male HIV^+^ | 614/  17,834 | 51.7% (47.8%, 56%) | **28.4 % (24.9%, 32.1%)** | 230/  17,834 | 58.4% (50.3%, 65.4%) | **31.9%**  **(27.6 %, 36.5 %)** |

*AGYW = adolescent girls and young women; N_exp_ = Number of individuals in each exposure category; N_t_ = total number of individuals in the denominator.*

**Table J in S1 Text.** Unique and joint contributions of male partner HIV status and male partner perpetrated physical and/or sexual IPV to HIV status among adolescent girls and young women. We present crude and adjusted risk differences.

|  | N_exp_/N_t_ | Crude risk difference  (95% CI) | Adjusted risk difference*  (95% CI) | N_exp_/N_t_ | Crude risk difference  (95% CI) | Adjusted risk difference*  (95% CI) |
| --- | --- | --- | --- | --- | --- | --- |
| Female HIV prevalence | **No past year IPV** | | | **Past year IPV** | | |
| Male HIV^-^ | 13,003/  17,834 | Referent | Referent | 3,987/  17,834 | 0.7%  (0.1%, 1.2%) | **0.4 %**^**^  **(-0.1%, 0.9%)** |
| Male HIV^+^ | 614/  17,834 | 49.8% (45.8%, 54.2%) | **26.6 %**  **(23.0%, 30.4%)** | 230/  17,834 | 56.5%  (48.5%, 63.5%) | **30.1 %**  **(25.6%, 34.7%)** |

* Adjusted for women’s age (continuous), wealth quintile, women’s education (none, primary, secondary, higher), residence type (rural, urban), women’s lifetime number of sexual partners (1, 2, $\geq3$), survey identifier.

** Does not add up to 0.5% [2.3% minus 1.8%] due to rounding (Table J)

*N_exp_ = Number of individuals in each exposure category; N_t_ = total number of individuals in the denominator.*

Based on Table J in S1 Text:

- The expected joint effect under an additive model: E(RD_expected_) = 26.6% + 0.4% = 27.0%
- The observed joint effect under an additive model: E(RD_observed_) = 30.1%
- The difference between the expected and observed effects: E(RD_observed_) - E(RD_expected_) = 3.1%

Therefore, HIV risk in AGYW whose IPV perpetrator partner lives with HIV exceeds by 3 cases (per 100 women) the sum of unique effects of male HIV status and IPV, indicating the presence of a small additive effect measure modification.

To further check for the presence of additive effect measure modification, we calculate the Relative Excess Risk due to Interaction (RERI): RERI= RD_11_-RD_01_-RD_01_+1 = 0.301 -0.266 -0.004+1 = 1.03

Same methodology as above was used to conduct the sensitivity analysis among women of all ages (Table K in S1 Text) and excluding those AGYW who had two or more sex partners in the past year (Table L in S1 Text).

**Table K in S1 Text.** Unique and joint contributions of male partner HIV status and male partner perpetrated IPV to female HIV status among all women over the age of 15. We present crude and adjusted risk differences.

|  | N_exp_/N_t_ | Crude risk difference  (95% CI) | Adjusted risk difference*  (95% CI) | N_exp_/N_t_ | Crude risk difference  (95% CI) | Adjusted risk difference*  (95% CI) |
| --- | --- | --- | --- | --- | --- | --- |
| Female HIV prevalence | **No past year IPV** | | | **Past year IPV** | | |
| Male HIV^-^ | 46,267/ 65,152 | Referent | Referent | 13,666/ 65,152 | 0.6% (0.1%, 1.1%) | **0.4 %**  **(0%, 0.9%)** |
| Male HIV^+^ | 4,082/ 65,152 | 48.5% (45.1%, 52.5%) | **22.1% (18.7%, 25.5%)** | 1,137/  65,152 | 48.8% (41.8%, 56.0%) | **19.4% (15.0%, 24.1%)** |

* Adjusted for women’s age (five-year age groups), wealth quintile, women’s education (none, primary, secondary, higher), residence type (rural, urban), women’s lifetime number of sexual partners (1, 2, $\geq3$), survey identifier.

*N_exp_ = Number of individuals in each exposure category; N_t_ = total number of individuals in the denominator.*

**Table L in S1 Text.** Unique and joint contributions of male partner HIV status and male partner perpetrated physical and/or sexual IPV to HIV status among adolescent girls and young women, *excluding women who had two or more sexual partners in the past year*. We present crude and adjusted risk differences.

|  | N_exp_/N_t_ | Crude risk difference  (95% CI) | Adjusted risk difference*  (95% CI) | N_exp_/N_t_ | Crude risk difference  (95% CI) | Adjusted risk difference*  (95% CI) |
| --- | --- | --- | --- | --- | --- | --- |
| Female HIV prevalence | **No past year IPV** | | | **Past year IPV** | | |
| Male HIV^-^ | 12,779/ 17,493 | Referent | Referent | 3,891/ 17,493 | 0.7%  (0.2%, 1.3%) | **0.5 %**  **(-0.1%, 1%)** |
| Male HIV^+^ | 598/ 17,493 | 49.5% (45.7%, 53.2%) | **26.7 %**  **(23.0%, 30.7%)** | 225/  17,493 | 56.7%  (49.8%, 63.6%) | **30.2 %**  **(25.2%, 35.4%)** |

* Adjusted for women’s age (continuous), wealth quintile, women’s education (none, primary, secondary, higher), residence type (rural, urban), women’s lifetime number of sexual partners (1, 2, $\geq3$), survey identifier.

*N_exp_ = Number of individuals in each exposure category; N_t_ = total number of individuals in the denominator.*

**Text B. Sensitivity analysis for the effects of selection bias on male HIV seroprevalence analysis.**

**Methods**

We conducted probabilistic sensitivity analysis to estimate the effects of selection bias on the association between the perpetration of past year IPV and male HIV prevalence. We followed the steps below per Lash et al. [2]

1. Identify the selection probabilities ($\alpha$, $\beta$, $\gamma$ and $\delta)$ based on the published literature (Table M in S1 Text).

As the baseline bias parameter among men who are HIV negative and do not perpetrate IPV *(*$\delta)$*,* we used the mean HIV testing response rates since all participants would have to have consented to HIV testing to be included in this analysis. This value is similar to the median HIV testing response rates among men (male response rate: 77.1%).[3] For the bias parameter for men who are HIV negative and do perpetrate IPV ($\gamma)$, we assumed a 5% reduction in response rate. A paper by Barnighausen shows that those who are living with HIV were four times as likely to refuse participation in HIV testing, compared to those who were HIV negative, suggesting that nonparticipation is associated with HIV status.[4] Therefore, we scaled down the bias parameters for men living with HIV ($\alpha$, $\beta$) by four compared to the respective bias parameters among men not living with HIV ($\gamma$*,* $\delta$*)*

**Table M in S1 Text.** Bias parameter values and data sources used for the selection bias sensitivity analysis.

| **Observed data** | **Bias parameter** | **Population** | **Bias parameter value** | **Bias parameter value data source** |
| --- | --- | --- | --- | --- |
| A° | $\alpha$ | IPV^+^, HIV^+^ | 0.190 | Postulate that men living with HIV are four times less likely to participate in HIV testing, compared to those not living with HIV ($\gamma$/4)[4] |
| B° | $\beta$ | IPV^-^, HIV^+^ | 0.199 | Postulate that men living with HIV are four times less likely to participate in HIV testing, compared to those not living with HIV ($\delta/4)$ [4] |
| C° | $\gamma$ | IPV^+^, HIV^-^ | 0.758 | Assume a 5% reduction in response rates as compared to $\delta$ |
| D° | $\delta$ | IPV^-^, HIV^-^ | 0.798 | Average HIV testing response rate[5] |

*IPV^+^ = Perpetrated IPV in the past year; IPV^-^ = Did not perpetrate IPV in the past year.*

*HIV^+^ = living with HIV; HIV^-^ = not living with HIV.*

1. Continuously resample a random value from a uniform probability distribution built around these bias parameters. The distribution bounds were built by increasing or decreasing the selection probabilities by 15%.
2. Use simple bias analysis to correct the prevalence ratio using the formula (1) where $\alpha$, $\beta$, $\gamma$ and $\delta$ are selection probabilities and $A^{\circ}$, $B^{\circ}$, $C^{\circ}$, $D^{\circ}$ are observed data. This gives us a systematic error-corrected estimate. [6]

PR_corrected_ = $\frac{(A^{\circ}/\alpha)}{( A^{\circ}/\alpha+ C^{\circ}/\gamma)}$/ $\frac{(B^{\circ}/\beta)}{( B^{\circ}/ \beta+ D^{\circ}/\delta)}$ (1)

1. To simulate an additional random error, choose a random standard normal deviate and multiply it by the standard error from the estimate of crude association between IPV and male HIV prevalence based on the observed data.
2. For each simulation combine the systematic and random error as follows:

Estimate _total_ = estimate _systematic_ – random_0,1_*ste_observed_

Here estimate_total_ is a single simulated estimate of association that incorporates both systematic and random error, estimate_systematic_ is a single simulated estimate corrected for only systematic error (from step 3), random _0,1_ is a random standard normal deviate, and ste_observed_ is a standard error from observed data.

As the “estimate _systematic_” is the ratio measure of association, we take its natural log and exponentiate formula (1) to get the final formula (2)

Estimate _total_ = $e^{\log\left( estimate_{systematic} \right)-random_{0,1}*ste_{observed}}$ (2)

1. Pool and summarize the estimates by calculating the median value. Calculate the 95% uncertainty intervals.

**Results:**

Based on the analysis above, the observed crude PR (cPR_obs_) = 0.98, while the bias corrected value is cPR_corrected_= 0.983 (95%CI: 0.757-1.260).

Given the similarity between the crude and bias corrected values, we do not anticipate a qualitatively significant impact of selection bias on the association between past year IPV perpetration and men’s HIV prevalence. However, we state this with caution given that our bias analysis is conditional on the provided bias parameters.

Keeping the bias parameters among men not living with HIV constant ($\gamma$ and $\delta$) and varying the proportion of survey participation among men living with HIV ($\alpha$ and $\beta)$ we show that, for the observed cPR to be an overestimate (cPR_obs_= 0.98), the survey response rates should be higher among IPV perpetrators than in IPV non-perpetrators ($\alpha$ > $\beta$) which is unlikely (Table N in S1 Text).[7]

**Table N in S1 Text.** Effect of various bias parameter values on corrected crude prevalence ratio.

| **Bias parameters** | | **cPR_corrected_** |
| --- | --- | --- |
| **IPV^+^, HIV^+^ (**$\boldsymbol{\alpha}$***)*** | **IPV^-^, HIV^+^ (**$\boldsymbol{\beta)}$ |  |
| 0.253 | 0.199 | 0.79 |
| 0.217 | 0.199 | 0.89 |
| 0.200 | 0.199 | 0.95 |
| 0.150 | 0.199 | 1.16 |
| 0.190 | 0.267 | 1.22 |
| 0.190 | 0.228 | 1.08 |
| 0.190 | 0.218 | 1.04 |
| 0.190 | 0.200 | 0.98 |

*IPV^+^ = Perpetrated IPV in the past year; IPV-^+^ = Did not perpetrate IPV in the past year.*

*HIV^+^ = living with HIV; HIV^-^ = is not living with HIV.*

**Text C: STROBE Statement—checklist of items that should be included in reports of observational studies**

|  | Item No. | Recommendation | | Page  No. | | | Relevant text from manuscript | | |
| --- | --- | --- | --- | --- | --- | --- | --- | --- | --- |
| **Title and abstract** | 1 | (*a*) Indicate the study’s design with a commonly used term in the title or the abstract | | 2 | | | “We pooled individual-level data from nationally representative, cross-sectional surveys from 27 countries in Africa (2000-2020).” | | |
|  |  | (*b*) Provide in the abstract an informative and balanced summary of what was done and what was found | | 2 | | | Abstract, Paragraph 2-3 | | |
| Introduction | | | | | |  | | |  |
| Background/rationale | 2 | Explain the scientific background and rationale for the investigation being reported | | 5  4 | | | **Rationale:** “Improving understanding of the factors and pathways associated with male-perpetrated IPV and their implications for women’s HIV acquisition risk is important to meet this commitment”; Introduction, Paragraph 1  **Scientific background**: Introduction, Paragraph 2 and 3. | | |
| Objectives | 3 | State specific objectives, including any prespecified hypotheses | | 5 | | | “The aim of this study is to describe the characteristics of men perpetrating physical and/or sexual IPV and investigate how these characteristics impact women’s HIV status among cohabiting couples in […] Specifically, we address three research questions. First, what male partner and partnership-level characteristics are associated with IPV? Second, are men who are reported to perpetrate IPV more likely to report behaviors that increase their risk of HIV acquisition and to be living with HIV? Third, does experiencing IPV increase young women’s risk of living with HIV, beyond the risk associated with their male partner’s HIV status? “ | | |
| Methods | | | | | |  | | |  |
| Study design | 4 | Present key elements of study design early in the paper | 6 | | “We reviewed available nationally representative, cross-sectional surveys conducted in 27 countries in Africa between 2000 and 2020 with available respondent-level data on IPV and HIV.” | | |  |  |
| Setting | 5 | Describe the setting, locations, and relevant dates, including periods of recruitment, exposure, follow-up, and data collection | 6 | | **Setting and timeline:** Methods, Paragraph 1 | | |  |  |
| Participants | 6 | (*a*) *Cohort study*—Give the eligibility criteria, and the sources and methods of selection of participants. Describe methods of follow-up  *Case-control study*—Give the eligibility criteria, and the sources and methods of case ascertainment and control selection. Give the rationale for the choice of cases and controls  *Cross-sectional study*—Give the eligibility criteria, and the sources and methods of selection of participants | 6 | | **Eligibility and sampling:** “The study population comprised currently cohabiting, married or partnered women and men (≥15 years) that participated in the *Demographic and Health Surveys* (DHS), *AIDS Indicator Survey* (AIS), and *Population-based HIV Impact Assessment* (PHIA) surveys. […] In PHIA, data on past-year IPV were collected from one randomly selected woman in each household and, in DHS, from all women in a fraction of households (usually one third).” | | |  |  |
|  |  | (*b*) *Cohort study*—For matched studies, give matching criteria and number of exposed and unexposed  *Case-control study*—For matched studies, give matching criteria and the number of controls per case | NA | | - | | |  |  |
| Variables | 7 | Clearly define all outcomes, exposures, predictors, potential confounders, and effect modifiers. Give diagnostic criteria, if applicable | 6-7  8-9 | | **Exposures:** Perpetration of physical and/or sexual IPV over the past year among cohabitating couples was defined based on the women’s self-reported experience of IPV, which was defined as the experience of physical and/or sexual violence in the past year by a current or former male intimate partner in the context of marriage or cohabitation. Current partners of women experiencing IPV in the past year were assumed to be perpetrators of IPV.    **Predictors:** “Potential factors correlated with IPV pertained to male individual factors and partnership-level factors. Individual factors included: accepting attitudes on IPV, alcohol use frequency, polygyny defined as having more than one wife/cohabiting partner. Partnership factors included: couple age and earning disparity, women’s say in household decision-making, and household headship (male/female) [….] Self-reported factors for men’s risk of living with HIV include: payment for sex in the past year, condom use at last sex with the most recent partner in the past year, number of sex partners in the past year, and point-prevalence of concurrency defined as having more than one sexual partnership at a single point in time six months before the interview. Definition of concurrency was aligned with the primary indicator recommended by the UNAIDS Reference Group on Estimates, Modelling and Projections Working Group on Measuring Concurrent Sexual Partnerships.  **Outcomes:** “HIV seropositivity was measured among consenting male and female participants at the time of survey administration via enzyme-linked immunosorbent assay (ELISA). The Zambia 2013-14 DHS was excluded from all analyses using HIV seropositivity due to a concern about the reliability of the HIV testing algorithm assay.”  **Potential confounders:** “Multivariable models were adjusted for basic socio-demographic variables: male age (five-year age groups to account for the non-linear age effect), household wealth quintiles and residence type (rural, urban), and male education (none, primary, secondary, higher). Survey-level fixed effects were included in the adjusted models to account for unmeasured survey-level confounders.”  “[…] The model was adjusted for female demographic characteristics (linear age effect for analysis specific to 15-24 year-old women, and five-year age groups in all women), household wealth and residence, education, women’s lifetime number of sex partners (1, 2, 3+) and survey-level fixed effects.” | | |  |  |
| Data sources/ measurement | 8* | For each variable of interest, give sources of data and details of methods of assessment (measurement). Describe comparability of assessment methods if there is more than one group | 6-7 | | **Exposure measurement:** “Current partners of women experiencing IPV in the past year were assumed to be perpetrators of IPV. From here onwards, when referring to “perpetrators” of IPV, we refer to men whose female partner reported experiencing IPV in the past year.”  **Outcome measurement:** “HIV seropositivity was measured among consenting male and female participants at the time of survey administration via enzyme-linked immunosorbent assay (ELISA). The Zambia 2013-14 DHS was excluded from all analyses using HIV seropositivity due to a concern about the reliability of the HIV testing algorithm assay.” | | |  |  |
| Bias | 9 | Describe any efforts to address potential sources of bias | 11  25 | | **Sensitivity analyses:** “Survey participation could be associated with both IPV and HIV status in men, leading to selection bias. In a probabilistic sensitivity analysis, we assumed selection probabilities which were assigned to perpetrators and non-perpetrators with and without the outcome of interest (HIV seropositivity) based on the existing literature.”  **Limitations:** Discussion, Paragraph 4 | | |  |  |
| Study size | 10 | Explain how the study size was arrived at | NA | | Existing survey data with a fixed sample size were used | | |  |  |

Continued on next page

| Quantitative variables | 11 | Explain how quantitative variables were handled in the analyses. If applicable, describe which groupings were chosen and why | 8-9 | **Groupings for continuous variables:**  Number of sex partners: “[…] women’s lifetime number of sex partners (1, 2, 3+) […]”  Age: “[…] male age (five-year age groups to account for the non-linear age effect) […]” |
| --- | --- | --- | --- | --- |
| Statistical methods | 12 | (*a*) Describe all statistical methods, including those used to control for confounding | 8-9 | **Adjustment for potential confounders:** “Multivariable models were adjusted for basic socio-demographic variables: male age (five-year age groups to account for the non-linear age effect), household wealth quintiles and residence type (rural, urban), and male education (none, primary, secondary, higher). Survey-level fixed effects were included in the adjusted models to account for unmeasured survey-level confounders.”  “The model was adjusted for female demographic characteristics (linear age effect for analysis specific to 15-24 year-old women, and five-year age groups in all women), household wealth and residence, education, women’s lifetime number of sex partners (1, 2, 3+) and survey-level fixed effects”  **Statistical models used**: “We used univariable Poisson regression models based on Generalized Estimating Equations (GEE) with robust standard errors and clustering by primary sampling unit (PSU).”  “We used marginal standardization based on GEE with robust standard errors.” |
|  |  | (*b*) Describe any methods used to examine subgroups and interactions | 9 | **Subgroup analysis and justification:** “We restricted this analysis to adolescent girls and young women aged 15-24 years for two reasons. First, we aimed to estimate the additional HIV risk due to IPV in the subgroup of women with the highest IPV prevalence and HIV incidence. Second, older women are more likely to have lived with HIV for longer due to higher HIV incidence in younger age groups. Therefore, past-year IPV is more likely to have preceded HIV acquisition among women aged 15-24 years.”  **Addressing effect measure modification:** “To quantify the magnitude of EMM under an additive model, we calculated the difference between the expected joint effect of male HIV status and IPV perpetration (the sum of their unique effects) and their observed joint effects.” |
|  |  | (*c*) Explain how missing data were addressed | 12 | “Eighteen surveys were excluded due to physical IPV questions not asked, IPV data missingness, or women in couples’ dataset not selected for the IPV module.” |
|  |  | (*d*) *Cohort study*—If applicable, explain how loss to follow-up was addressed  *Case-control study*—If applicable, explain how matching of cases and controls was addressed  *Cross-sectional study*—If applicable, describe analytical methods taking account of sampling strategy | 8 | “We used Generalized Estimating Equations (GEE) with robust standard errors and clustering by primary sampling unit (PSU), without survey weights.” |
|  |  | (*e*) Describe any sensitivity analyses | 10-11 | **Sensitivity analyses:** “First, we explored the heterogeneity of effect size estimates across survey for each model by calculating survey-specific crude prevalence ratios and pooling them using both fixed and random-effect meta-analyses. We conducted subgroup (moderator) analyses by survey region and/or year when heterogeneity was moderate (25%-50%) to high (>50%). Second, we also calculated crude and adjusted prevalence ratios stratified by region. Third, we excluded women who had two or more sexual partners in the past year to reduce the likelihood that women’s reports of experiencing IPV in the past year refers to someone other than their current, cohabiting partner.” |
| Results | | | | |
| Participants | 13* | (a) Report numbers of individuals at each stage of study—eg numbers potentially eligible, examined for eligibility, confirmed eligible, included in the study, completing follow-up, and analysed | 11 | **Description of included surveys and the study population**: Results, Paragraph 1 |
|  |  | (b) Give reasons for non-participation at each stage | 14 | Figure 1 |
|  |  | (c) Consider use of a flow diagram | 14 | Figure 1 |
| Descriptive data | 14* | (a) Give characteristics of study participants (eg demographic, clinical, social) and information on exposures and potential confounders | 12 | **Characteristics of the study population:** Results, Paragraph 2 and 3 |
|  |  | (b) Indicate number of participants with missing data for each variable of interest | 15 | Table 1 |
|  |  | (c) *Cohort study*—Summarise follow-up time (eg, average and total amount) | NA | *-* |
| Outcome data | 15* | *Cohort study*—Report numbers of outcome events or summary measures over time | NA | *-* |
|  |  | *Case-control study—*Report numbers in each exposure category, or summary measures of exposure | NA | *-* |
|  |  | *Cross-sectional study—*Report numbers of outcome events or summary measures | 12-13 | Results, Paragraph 2 and 3 |
| Main results | 16 | (*a*) Give unadjusted estimates and, if applicable, confounder-adjusted estimates and their precision (eg, 95% confidence interval). Make clear which confounders were adjusted for and why they were included |  | Table 2, Table 3, Figure 2  We report both unadjusted and adjusted estimates with relevant confidence intervals throughout the Results section. Table 1 and 2 footnotes include a full list of confounders that were adjusted for. |
|  |  | (*b*) Report category boundaries when continuous variables were categorized | 9  8 | Number of sex partners: “[…] women’s lifetime number of sex partners (1, 2, 3+) […]”  Age: “[…] male age (five-year age groups to account for the non-linear age effect) […]” |
|  |  | (*c*) If relevant, consider translating estimates of relative risk into absolute risk for a meaningful time period | 19, 20,21 | **The role of male-perpetrated physical and/or sexual intimate partner violence in adolescent girls and young women’s risk of HIV seroprevalence:** Throughout our third objective we are using absolute (risk difference), as opposed to relative measures. |

Continued on next page

| Other analyses | 17 | Report other analyses done—eg analyses of subgroups and interactions, and sensitivity analyses | 19  22 | “Sensitivity analysis (Text 2, S1 Appendix) does not indicate a noteworthy impact of selection bias on the association between past year perpetration of IPV and male HIV seroprevalence.“  “Sensitivity analyses show that the removal of women who had two or more sex partners in the past year from our analyses does not change our estimates of the contribution of IPV in AGYW’s risk of HIV seroprevalence (Table L, S1 Appendix).“ |
| --- | --- | --- | --- | --- |
| Discussion | | | | |
| Key results | 18 | Summarise key results with reference to study objectives | 23 | “Pooling data from 48 surveys from 27 countries in Africa, including up to 111,600 couples, we found that men whose partners reported that they perpetrate IPV are more likely to share behaviors that increased their risk of HIV acquisition and transmission than men who do not. They are also more likely to be living with HIV. Further, AGYW whose male partners perpetrate IPV have a small (3%) added risk of living with HIV in addition to the risk entailed solely by their partners’ HIV status.“ |
| Limitations | 19 | Discuss limitations of the study, taking into account sources of potential bias or imprecision. Discuss both direction and magnitude of any potential bias | 24-25 | Discussion, paragraph 4 |
| Interpretation | 20 | Give a cautious overall interpretation of results considering objectives, limitations, multiplicity of analyses, results from similar studies, and other relevant evidence | 26 | “Ending IPV may not single-handedly eliminate HIV acquisition in women since the added risk of living with HIV due to IPV, beyond the risk entailed solely in their partners’ HIV status could be small. Still, experiencing IPV adds to AGYW’s risk of living with HIV, which demonstrates the mutually reinforcing effects of HIV/IPV and the importance of addressing both issues simultaneously.” |
| Generalisability | 21 | Discuss the generalisability (external validity) of the study results | 26-27 | Discussion, final paragraph |
| Other information | |  | | |
| Funding | 22 | Give the source of funding and the role of the funders for the present study and, if applicable, for the original study on which the present article is based | NA | We have included the funding information in the “Sources of funding” section of the manuscript submission form. |

*Give information separately for cases and controls in case-control studies and, if applicable, for exposed and unexposed groups in cohort and cross-sectional studies.

**Note:** An Explanation and Elaboration article discusses each checklist item and gives methodological background and published examples of transparent reporting. The STROBE checklist is best used in conjunction with this article (freely available on the Web sites of PLoS Medicine at http://www.plosmedicine.org/, Annals of Internal Medicine at http://www.annals.org/, and Epidemiology at http://www.epidem.com/). Information on the STROBE Initiative is available at www.strobe-statement.org.

**References**

1. Muller CJ, MacLehose RF. Estimating predicted probabilities from logistic regression: different methods correspond to different target populations. Int J Epidemiol. 2014;43(3):962-70. DOI:10.1093/ije/dyu029.

2. Lash TL, Fox MP, Fink AK. Applying quantitative bias analysis to epidemiologic data. Dordrecht ; New York: Springer; 2009.

3. Mishra V, Hong W, Khan S, Gu Y, Liu L. Evaluating HIV Estimates from National Population-Based Surveys for Bias Resulting from Non-Response 2008. Available from:

4. Barnighausen T, Bor J, Wandira-Kazibwe S, Canning D. Correcting HIV prevalence estimates for survey nonparticipation using Heckman-type selection models. Epidemiology. 2011;22(1):27-35. DOI:10.1097/EDE.0b013e3181ffa201.

5. Marino M, Pagano M. Role of survey response rates on valid inference: an application to HIV prevalence estimates. Emerg Themes Epidemiol. 2018;15:6. DOI:10.1186/s12982-018-0074-x.

6. Kleinbaum DG, Sullivan KM, Barker ND. ActivEpi companion textbook : a supplement for use with the ActivEpi CD-ROM. Second edition. ed. New York: Springer; 2013.

7. Skafida V, Devaney J. Answer Refused: Exploring How Item Non-response on Domestic Abuse Questions in a Social Survey Affects Analysis. Journal of European Survey Research Association. 2022;16(2).
